# Cardiomyocyte GTP Cyclohydrolase 1 Protects the Heart Against Diabetic Cardiomyopathy

**DOI:** 10.1038/srep27925

**Published:** 2016-06-13

**Authors:** Hsiang-En Wu, Shelley L. Baumgardt, Juan Fang, Mark Paterson, Yanan Liu, Jianhai Du, Yang Shi, Shigang Qiao, Zeljko J. Bosnjak, David C. Warltier, Judy R. Kersten, Zhi-Dong Ge

**Affiliations:** 1 Department of Anesthesiology, Medical College of Wisconsin, 8701 Watertown Plank Road, Milwaukee, WI 53226, USA; 2 National Institute on Drug Abuse, National Institutes of Health, 251 Bayview Boulevard, Baltimore, MA 21224, USA; 3 Department of Pediatrics, Medical College of Wisconsin, 8701 Watertown Plank Road, Milwaukee, WI 53226, USA; 4 Department of Medicine, Columbia University, 630 W. 168th Street, New York, NY 10032, USA; 5 Department of Biochemistry, University of Washington, 1705 NE Pacific Street, Seattle, WA 98195, USA; 6 Aurora Research Institute, Aurora Health Care, 750 W. Virginia Street, Milwaukee, WI 53234, USA; 7 Department of Physiology, Medical College of Wisconsin, 8701 Watertown Plank Road, Milwaukee, WI 53226, USA; 8 Department of Pharmacology and Toxicology, Medical College of Wisconsin, 8701 Watertown Plank Road, Milwaukee, WI 53226, USA

## Abstract

Diabetic cardiomyopathy increases the risk of heart failure and death. At present, there are no effective approaches to preventing its development in the clinic. Here we report that reduction of cardiac GTP cyclohydrolase 1 (GCH1) degradation by genetic and pharmacological approaches protects the heart against diabetic cardiomyopathy. Diabetic cardiomyopathy was induced in C57BL/6 wild-type mice and transgenic mice with cardiomyocyte-specific overexpression of GCH1 with streptozotocin, and control animals were given citrate buffer. We found that diabetes-induced degradation of cardiac GCH1 proteins contributed to adverse cardiac remodeling and dysfunction in C57BL/6 mice, concomitant with decreases in tetrahydrobiopterin, dimeric and phosphorylated neuronal nitric oxide synthase, sarcoplasmic reticulum Ca^2+^ handling proteins, intracellular [Ca^2+^]_i_, and sarcoplasmic reticulum Ca^2+^ content and increases in phosphorylated p-38 mitogen-activated protein kinase and superoxide production. Interestingly, GCH-1 overexpression abrogated these detrimental effects of diabetes. Furthermore, we found that MG 132, an inhibitor for 26S proteasome, preserved cardiac GCH1 proteins and ameliorated cardiac remodeling and dysfunction during diabetes. This study deepens our understanding of impaired cardiac function in diabetes, identifies GCH1 as a modulator of cardiac remodeling and function, and reveals a new therapeutic target for diabetic cardiomyopathy.

The incidence of type 1 diabetes mellitus (T1DM) has been increasing by 2% to 5% annually worldwide^1^. Although treatment of diabetes has substantially improved in recent decades, a higher rate of cardiac dysfunction occurs in patients with T1DM compared with nondiabetics^2^,^3^,^4^,^5^. This cardiac dysfunction occurs in T1DM patients without a recognized cause such as coronary artery disease or hypertension, termed diabetic cardiomyopathy (DCM)^4^,^6^,^7^. Clinical studies indicate that DCM increases the risk of heart failure and death^8^,^9^,^10^,^11^,^12^. However, there are no effective approaches to preventing the development and progression of DCM in the clinic^13^,^14^. The search for new therapeutic targets for protection of diabetic hearts is of primary importance.

Intracellular free Ca^2+^ ([Ca^2+^]_i_) is a primary determinant of contraction and relaxation of cardiac muscle^15^,^16^. In cardiomyocytes, the sarcoplasmic reticulum (SR) Ca^2+^ release channels, ryanodine receptors (RyR2), control Ca^2+^ release from the SR to trigger muscle contraction, whereas the SR Ca^2+^ ATPase (SERCA2a) removes Ca^2+^ from the cytosol to induce relaxation^15^. Previous studies suggest that SR Ca^2+^ release and subsequent re-uptake into the SR, called SR Ca^2+^ cycling, are depressed by diabetes in rat cardiomyocytes^17^. These impairments in SR Ca^2+^ cycling are putatively considered as the critical cause of DCM^18^. Recent studies suggest that cardiomyocyte GTP cyclohydrolase 1 (GCH1) favorably regulates SR Ca^2+^ cycling in normal myocardium^19^. However, GCH1 proteins in vascular endothelium are found to decrease during diabetes and hyperglycemia^20^. It remains unknown how cardiomyocyte GCH1 is modulated in DCM, and whether cardiomyocyte-targeted increase of GCH1 proteins benefits intracellular Ca^2+^ signaling, thus improving cardiac function in diabetes.

GCH1 is the first and rate-limiting enzyme in the *de novo* biosynthesis of tetrahydrobiopterin (BH_4_), an essential co-factor for all 3 isoforms of nitric oxide synthase (NOS): neuronal NOS (nNOS), inducible NOS (iNOS), and endothelial NOS (eNOS)^21^. When BH_4_ is adequate, NOS proteins form homodimers, which oxidizes the substrate L-arginine to produce nitric oxide (NO), termed NOS coupling^22^. Previous studies suggest that deficiency of BH_4_ causes dimeric NOS proteins to become monomeric, which is unable to oxidize L-arginine to produce NO^22^,^23^. Instead, molecular oxygen is reduced to form superoxide (O_2_^•−^)^24^. This phenomenon is known as NOS uncoupling. A growing body of evidence suggests that NOS uncoupling is involved in cardiac dysfunction and the pathogenesis of DCM^25^,^26^,^27^. Whether cardiomyocyte-targeted increase of GCH1 proteins is capable of preventing NOS uncoupling in diabetes remains unclear.

P38 MAPK is the member of the MAPK family that is activated (phosphorylated) by a variety of environmental stressors and inflammatory cytokines^28^. Recent studies suggest that phosphorylated p38 (p-p38) MAPK contributes to the pathogenesis of cardiomyopathy including DCM^29^,^30^. Immunoprecipitation and proteomic analysis reveal that GCH1 forms protein complexes with p38 MAPK within cells^31^. Whether there is an interaction between cardiomyocyte GCH1 and p38 MAPK during diabetes is unknown.

In this study, we examined the regulation, function, and therapeutic potential of cardiac GCH1 in DCM. We found that T1DM caused the degradation of cardiac GCH1 proteins and impaired cardiac function in C57BL/6 wild-type (WT) mice. To increase cardiomyocyte GCH1, we generated a transgenic (Tg) mouse with cardiomyocyte-specific overexpression of GCH1 under the control of the α-myosin heavy chain promoter^32^. In this unique mouse model, GCH1 was elevated specifically in cardiomyocytes rather than coronary vascular endothelial cells, cardiac fibroblasts, or coronary vascular smooth muscle cells^19^,^33^. To study molecular mechanisms linking GCH1 with cardiac function, we determined GCH1, BH_4_, NOS, p38 MAPK, SR Ca^2+^ handling proteins, and intracellular Ca^2+^ signaling in WT and Tg mice with or without DCM. We hypothesized that cardiomyocyte GCH1 attenuates cardiac remodeling and dysfunction via up-regulation of BH_4_, nNOS, and SR Ca^2+^ handling proteins and suppression of p38 MAPK in DCM^19^,^31^. Lastly, we used MG 132 (a 26S proteasome inhibitor) to prevent the degradation of cardiac GCH1 in diabetic WT mice to test the potential of GCH1 as a therapeutic target on DCM.

## Results

### GCH1 is obligatory for cardiac function

**Figure 1:**
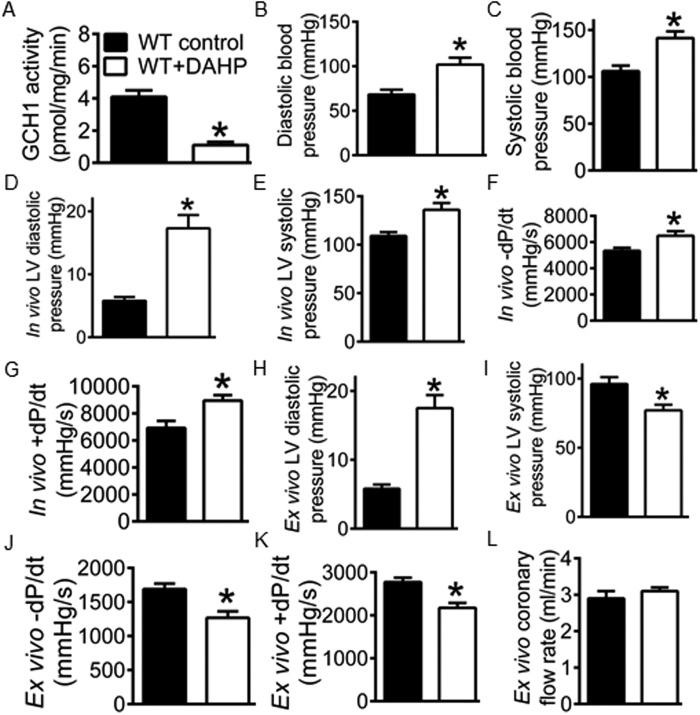
Inhibition of GTP cyclohydrolase 1 (GCH1) attenuates cardiac function in isolated hearts of C57BL/6 wild-type (WT) mice. (**A**) reduced GCH-1 activity by 2,4-diamino-6-hydroxy-pyrimidine (DAHP); (**B**) elevated diastolic blood pressure by DAHP; (**C**) elevated systolic blood pressure by DAHP; (**D**) increased *in vivo* left ventricular diastolic pressure by DAHP; (**E**) elevated *in vivo* left ventricular systolic pressure by DAHP; (**F**) increased *in vivo* −dP/dt by DAHP; (**G**) increased *in vivo* + dP/dt by DAHP; (**H**) increased *ex vivo* diastolic pressure by DAHP; (**I**) decreased *ex vivo* systolic pressure by DAHP; (**J**) decreased *ex vivo* -dP/dt by DAHP; (**K**) decreased *ex vivo* + dP/dt by DAHP; (**L**) *ex vivo* coronary flow rate. From panel B to panel G, blood pressure and left ventricular pressure was determined by a pressure catheter placed within the right carotid artery and the chamber of the left ventricle (LV) in intact mice. From panel H to panel L, cardiac function and coronary flow rate were measured in isolated Langendorff-perfused hearts. ^*^P < 0.05 versus WT controls (n = 10 mice/group).

To determine whether GCH1 is important for cardiac function, we used the specific inhibitor of GCH1, 2,4-diamino-6-hydroxy-pyridine (DAHP)^34^, to treat C57BL/6 WT mice and measured blood pressure, *in vivo* left ventricular pressure, and *in vivo* and *ex vivo* cardiac function. After the animals were orally given DAHP for 4 weeks^34^, cardiac GCH1 activity was significantly decreased in WT + DAHP group compared with WT control (Fig. 1A). A pressure-tipped catheter was inserted into the right carotid artery and subsequently the chamber of the left ventricle (LV) to measure the pressure^35^. Diastolic and systolic blood pressure, i*n vivo* LV systolic pressure and diastolic pressure were significantly elevated in WT + DAHP groups compared with WT controls (P < 0.05, n = 10 mice/group) (Fig. 1B–E). The values of +dP/dt (an indicator of systolic function) and −dP/dt (an indicator of diastolic function) calculated from contraction and relaxation of the LV were significantly greater in WT + DAHP than WT control groups (Fig. 1F,G). Hypertension is well known to affect cardiac function^36^,^37^. To study the direct effect of GCH1 inhibition on the heart, we measured cardiac function in isolated Langendorff-perfused hearts, as described^38^,^39^. The LV diastolic pressure in Langendorff-perfused hearts was adjusted to the level of the *in vivo* LV pressure (5.8 ± 0.6 mmHg in WT control and 17.5 ± 1.9 mmHg in WT + DAHP group) (Fig. 1D,H). The *ex vivo* LV systolic pressure was significantly decreased in WT + DAHP group compared with WT control (P < 0.05) (Fig. 1I). The values of ±dP/dt were significantly smaller in WT + DAHP than WT control groups (Fig. 1J,K). Coronary flow rate was not altered by DAHP (Fig. 1L). These results suggest that cardiac GCH1 is necessary for cardiac function in the mouse.

### Diabetes results in degradation of cardiac GCH1 proteins in WT mice

**Figure 2:**
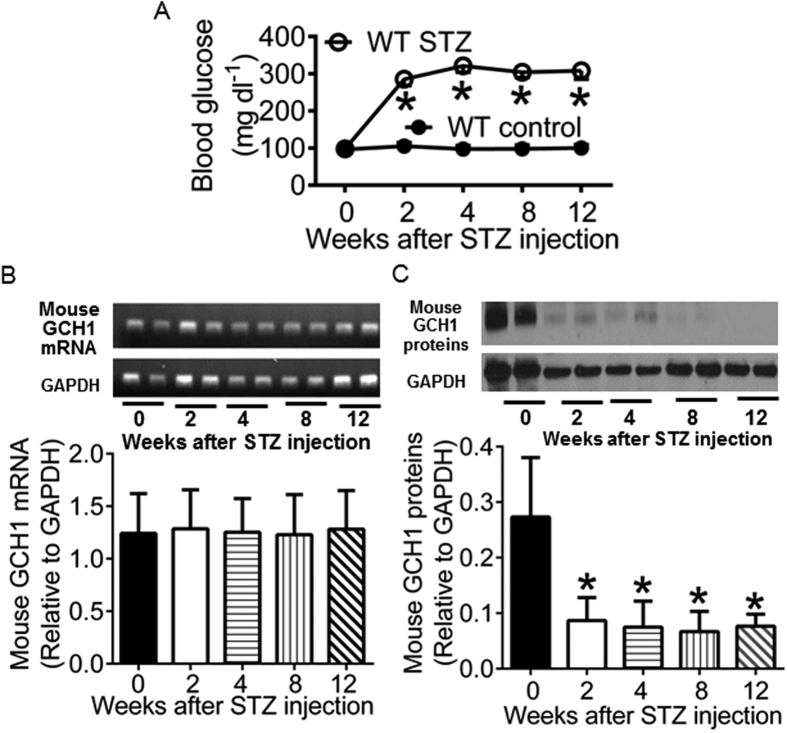
Streptozotocin (STZ)-induced diabetes reduces cardiac GTP cyclohydrolase 1 (GCH1) proteins in wild-type (WT) mice. (**A**) fasting blood glucose. ^*^P < 0.05 versus WT controls (n = 12 mice/group); (**B**) Mouse GCH1 mRNA levels. Top panel: representative PCR bands showing cardiac mouse GCH1 mRNA and glyceraldehyde 3-phosphate dehydrogenase (GAPDH) house-keeping gene 0, 2, 4, 8, 12 weeks after injection of STZ. Bottom panel: mouse GCH1 mRNA levels normalized to GAPDH (n = 6 mice/group); (**C**) Mouse GCH1 protein expression. Top panel: representative Western blot bands showing the expression of cardiac mouse GCH1 and GAPDH proteins from 0 to 12 weeks after administration of STZ. Bottom panel: GCH1 proteins normalized to GAPDH. ^*^P < 0.05 versus 0 weeks after injection of STZ (n = 6 heart/group).

Streptozotocin (STZ) destroys pancreatic β-cells and is often used to induce T1DM of insulin deficiency in experimental animals^40^,^41^. We used STZ to induce diabetes in C57BL/6 WT mice (WT STZ). Control animals were given citrate buffer (WT control). Fasting blood glucose, GCH1 mRNA, and GCH1 proteins were measured at baseline (0 weeks after administration of STZ or citrate buffer) and 2, 4, 8, and 12 weeks after intraperitoneal injection of STZ or citrate buffer, as previously shown^42^. There were no significant differences in blood glucose, GCH1 mRNA, and GCH1 proteins between WT STZ and WT control groups at baseline (Fig. 2). Administration of STZ induced a dramatic increase in blood glucose levels but did not change the levels of GCH1 mRNA from 2 to 12 weeks after injection of STZ. Interestingly, the expression of GCH1 proteins was significantly decreased compared with baseline levels (P < 0.05, n = 6 mice/group). Since GCH1 mRNA levels were not changed, decreased GCH1 proteins by diabetes were not due to reduction of synthetic GCH1 rather than increased degradation of GCH1 proteins.

### GCH1 transgene drives GCH1 protein expression in non-diabetic and diabetic myocardium

**Figure 3:**
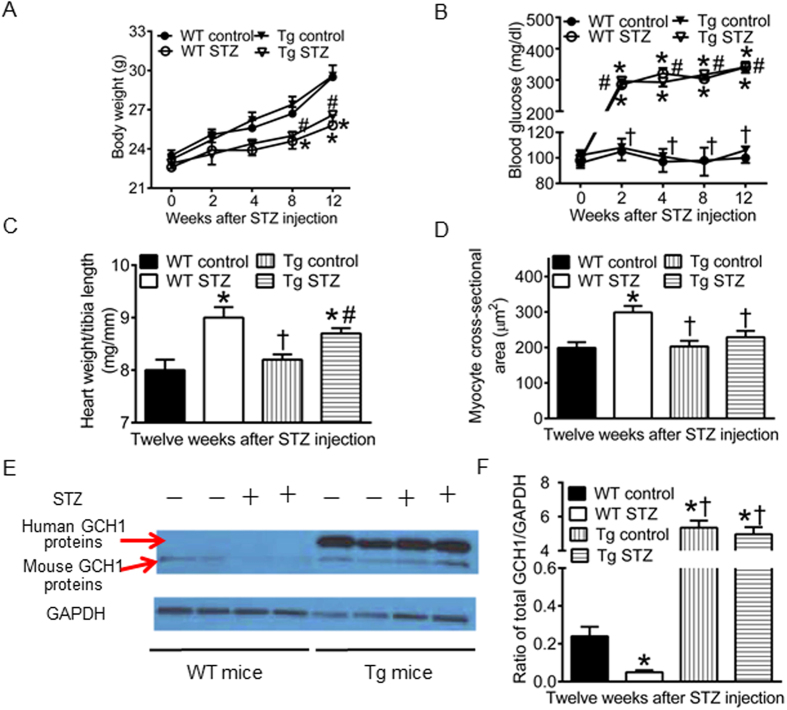
Human GTP cyclohydrolase 1 (GCH1) transgene drives GCH1 protein expression in both non-diabetic and diabetic myocardia. (**A**) time-dependent changes in body weight of wild-type (WT) and tranegenic (Tg) GCH1 mice after injection of streptozotocin (STZ) or vehicle (control) (n = 12 mice/group); (**B**) fasting blood glucose (n = 12 mice/group); (**C**) heart weight normalized to tibia length (n = 10 mice/group); (**D**) cardiomyocyte cross-sectional area (n = 6-7 mice/group); (**E**) representative Western blot bands showing the expression of human and mouse GCH1 proteins and glyceraldehyde 3-phosphate dehydrogenase (GAPDH) as a loading control; (**F**) the ratio of GCH1 proteins/GAPDH in WT and Tg mice 12 weeks after induction of diabetes (n = 4 mice/group). ^*^P < 0.05 versus WT controls; ^†^P < 0.05 versus WT STZ groups; ^#^P < 0.05 versus Tg controls.

To study how transgenic GCH1 impacts cardiac GCH1 proteins in diabetics, the Tg mice and their WT littermates were made diabetic with STZ (Tg STZ and WT STZ) or given citrate buffer as control (Tg control and WT control). There were no significant differences in body weight and fasting blood glucose among 4 experimental groups at baseline and between Tg control and WT control groups from 2 to 12 weeks after injection of the vehicle (Fig. 3). Compared with WT control groups, body weight was decreased in WT STZ and Tg STZ groups 8 and 12 weeks after induction of diabetes (Fig. 3A), and blood glucose was significantly increased in WT STZ and Tg STZ groups from 2 to 12 weeks after induction of diabetes (P < 0.05, n = 10 mice/group) (Fig. 3B). There were no significant differences in body weight and fasting blood glucose between Tg STZ and WT STZ groups throughout the experiment (P > 0.05). Heart weight normalized to tibia length was greater in WT STZ or Tg STZ than WT control groups 12 weeks after induction of diabetes (Fig. 3C). Cardiomyocyte cross-sectional areas measured from Masson’s trichrome-stained hearts were larger in WT STZ than WT control groups (Fig. 3D). There were no significant differences in myocyte cross-sectional area between Tg control and WT control or Tg STZ groups.

In the Tg mice, human GCH1 gene was transferred to mice^32^. We used Western blot to measure the expression of human and mouse GCH1 proteins in both diabetics and nondiabetics. In the Tg mice, both mouse and human GCH proteins were expressed in myocardium (Fig. 3E). Expression of human GCH1 proteins did not significantly alter mouse GCH1 proteins in the hearts of Tg control mice compared with WT control. Administration of STZ resulted in a significant decrease in the expression of GCH1 proteins in WT mice. Interestingly, transgenic overexpression of human GCH1 gene dramatically increased the expression of GCH1 proteins (mouse GCH1 proteins + human GCH1 proteins) not only in Tg control mice but also in Tg STZ mice compared with WT control (P < 0.05, n = 4 mice/group) (Fig. 3E,F). These results suggest that human GCH1 transgene drives GCH1 protein expression in both non-diabetic and diabetic myocardia.

### GCH1 overexpression ameliorates diabetes-induced cardiac remodeling and dysfunction

**Figure 4:**
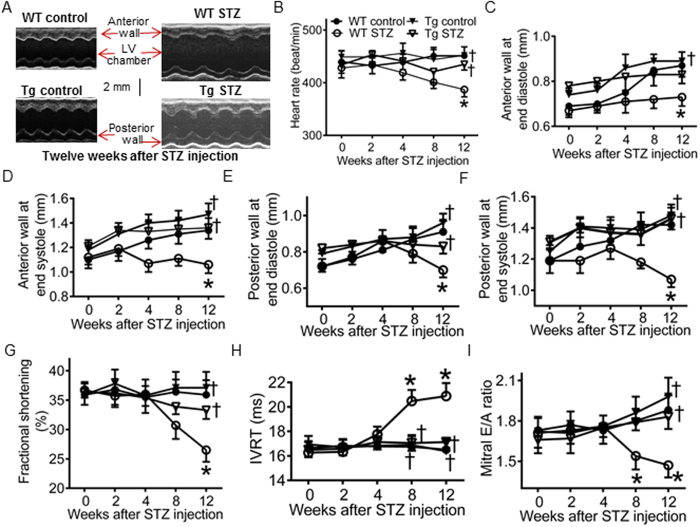
GTP cyclohydrolase 1 (GCH1) overexpression attenuates ardiac remodeling and dysfunction induced by diabetes. (**A**) representative M-mode echocardiograms of wild-type (WT) and transgenic (Tg) mice with or without diabetes. The scale bar represents 2 mm; (**B**) time-dependent changes in heart rate in WT and Tg mice after induction of diabetes with streptozotocin (STZ); (**C**) anterior wall thickness at end-diastole; (**D**) anterior wall thickness at end-systole; (**E**) posterior wall thickness at end diastole; (**F**) posterior wall thickness at end systole; (**G**) fractional shortening; (**H**) isovolumic relaxation time (IVRT); (**I**) mitral E/A ratio. The dimensions and function of the left ventricle was evaluated with echocardiography. ^*^P < 0.05 versus WT controls; ^†^P < 0.05 versus WT STZ mice (n = 10 mice/group).

DCM is characterized by cardiac (both diastolic and later systolic) dysfunction that occurs independently of coronary artery disease or hypertension^43^. Non-invasive transthoracic echocardiography is often used to evaluate cardiac function in patients with DCM^3^,^4^. We used an echocardiography specific for evaluation of mouse hearts following induction of diabetes^32^,^44^. As shown in Fig. 4, anterior and posterior wall thickness of the LV at end diastole and end systole, fractional shortening (an indicator of systolic function), isovolumic relaxation time (IVRT), and mitral E/A ratio (an indicator of diastolic function) were comparable among 4 groups: WT control, WT STZ, Tg control, and Tg STZ at baseline (0 week after injection of STZ) (P > 0.05, n = 10 mice/group). STZ-induced diabetes decreased the thickness of the LV wall, fractional shortening, and mitral E/A ratio and increased in IVRT in WT mice 12 weeks after induction of diabetes. These deleterious effects of diabetes were significantly attenuated by GCH1 overexpression (P < 0.05 between Tg STZ and WT STZ groups). These results indicate that GCH1 overexpression reduces diabetes-induced adverse remodeling and dysfunction of the LV.

### GCH1 overexpression improves intracellular Ca^2+^ signaling impaired by diabetics

**Figure 5:**
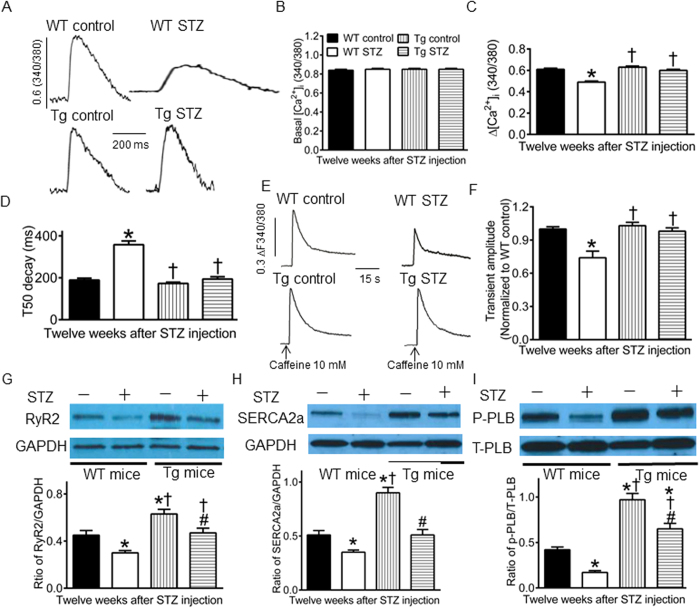
GTP cyclohydrolase 1 (GCH1) overexpression elevates intracellular [Ca^2+^ ]_i_, sarcoplasmic reticulum (SR) Ca^2+^ content, and expression of SR Ca^2+^ handling proteins decreased by diabetes. (**A**) original recordings of Ca^2+^ trantients in cardiomyocytes. The vertical scale bar indicates 0.6 fura-2 ratio (340/380 nm) unit, and the horizontal scale bar indicates 200 ms; (**B**) basal [Ca^2+^]_i_; (**C**) changes in intracellular [Ca^2+^]_i_; (**D**) time to half (T50) decay of the Ca^2+^ transient. From panel A to panel D, the cardiomyocytes isolated form diabetic (STZ) and nondiabetic (control) wild-type (WT) or transgenic (Tg) mice were loaded with fura-2 AM and electrically stimulated at 0.5 Hz. (**E**) original recordings of caffeine-induced SR Ca^2+^ release in cardiomyocytes in the presence of 0 Na^+^ and 0 Ca^2+^ Tyrode buffer. Arrows indicate that the application of 10 mM caffeine to cardiomyocytes to induce Ca^2+^ release; (**F**) caffeine-induced Ca^2+^ transient amplitude. *P < 0.05 versus WT control; ^†^P < 0.05 versus WT STZ group (n = 50–55 cells/group). (**G**) the ratio of ryanodine receptors 2 (RyR2)/glyceraldehyde 3-phosphate dehydrogenase (GAPDH). Top: representative Western blot bands of RyR2 and GAPDH in WT and Tg hearts. Bototm: the ratio of RyR2/GAPDH; (**H**) the ratio of SR Ca^2+^ ATPase (SERCA2a)/GAPDH. Top: representative Western blot bands of SERCA2a and GAPDH in WT and Tg hearts; (**I**) the ratio of phosphorylated phospholamban (p-PLB)/total phospholamban (T-PLB). *P < 0.05 versus WT controls, ^†^P < 0.05 versus WT STZ groups, ^#^P < 0.05 versus Tg controls (n = 4 hearts/group).

Abnormalities in intracellular Ca^2+^ signaling in cardiomyocytes are an important cause of DCM^18^. To study if GCH1 overexpression may reduce diabetes-induced impairments in intracellular Ca^2+^ signaling, we first measured intracellular [Ca^2+^]_i_ in fura-2-loaded cardiomyocytes isolated from Tg and WT mice with or without diabetes 12 weeks after administration of STZ or vehicle (Fig. 5A). There were no significant differences in basal [Ca^2+^]_i_ among 4 groups (Fig. 5B). Electric stimulation at 0.5 Hz evoked a significant Ca^2+^ transient in all cells. Compared with WT controls, changes in [Ca^2+^]_i_ (∆[Ca^2+^]_i_) was significantly decreased, and time to half (T50) decay of the Ca^2+^ transients was significantly prolonged in WT STZ groups (Fig. 5C,D). There were no significant differences in ∆[Ca^2+^]_i_ and T50 decay between Tg controls and WT controls, Tg STZ and WT controls, or Tg STZ and Tg controls (all P > 0.05).

Secondly, we studied the effect of GCH1 overexpression on SR Ca^2+^ content in diabetic cardiomyocytes. Caffeine was used to induce SR Ca^2+^ release in cardiomyocytes isolated from Tg and WT mice with or without diabetes in the presence of 0 Na^+^ and 0 Ca^2+^ Tyrode buffer^17^. Application of 10 mM caffeine to fura-2-loaded myocytes elicited a significant [Ca^2+^]_i_ transient in all 4 groups of mice (Fig. 5E). The amplitude of the caffeine-induced [Ca^2+^]_i_ transient was significantly lower in WT SZT mice than WT control mice (P < 0.05, n = 50–55 cells in 5 mice/group) (Fig. 5F). Interestingly, GCH1 overexpression significantly elevated the amplitude of the caffeine-induced [Ca^2+^]_i_ transient in diabetes compared with WT STZ group (P < 0.05 between Tg STZ and WT STZ groups). There were no significant differences in the caffeine-induced [Ca^2+^]_i_ transient between Tg STZ and Tg control or WT control (P > 0.05).

Thirdly, we determined the effects of GCH1 overexpression on the expression of SR Ca^2+^ handling proteins in diabetic hearts using Western blot analysis^44^,^45^. Compared with WT controls, GCH1 overexpression increased the ratios of RyR2/total phospholamban (T-PLB), SERCA2a/T-PLB, and phosphorylated phospholamban at serine 16 (p-PLB)/T-PLB (P < 0.05, n = 4 hearts/group) (Fig. 5G–I). Diabetes significantly decreased the ratios of RyR2/T-PLB, SERCA2a/T-PLB, and p-PLB/T-PLB in WT mice (P < 0.05 between WT STZ and WT controls) but not in Tg mice (P > 0.05 between Tg STZ and WT controls).

### Effects of GCH1 overexpression on BH_4_ and dimeric and phosphorylated NOS in diabetics

**Figure 6:**
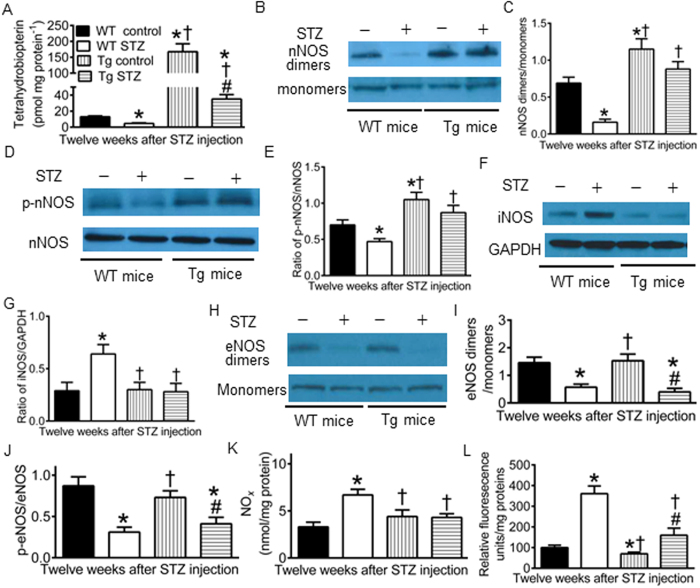
Effects of GCH1 overexpression on tetrahydrobiopterin, dimerization and phosphorylation of nitric oxide synthase (NOS), nitric oxide (NO_x_), and superoxide in diabetics. (**A**) tetrahydrobiopterin (BH_4_) concentrations (n = 5 mice/group); (**B**) representative Western blot bands showing the expression of dimers and monomers of neuronal NOS (nNOS) 12 weeks after administration of streptozotocin (STZ) or vehicle; (**C**) the ratio of nNOS dimers/monomers; (**D**) representative Western blot bands showing the expression of phosphorylated nNOS at serine 1412 (p-nNOS) and total nNOS; (**E**) the ratio of p-nNOS/total nNOS (n = 4 mice/group); (**F**) Western blot bands showing the expression of inducible NOS (iNOS) and glyceraldehyde 3-phosphate dehydrogenase (GAPDH); (**G**) the ratio of iNOS/GAPDH (n = 4 mice/group); (**H**) Western blot bands showing expression of the dimers and monomers of endothelial NOS (eNOS); (**I**) the ratio of eNOS dimers/monomers; (**J**) the ratio of phosphorylated eNOS at serine 1177 (p-eNOS)/total eNOS (n = 4 mice/group); (**K**) myocardial NO_*x*_ levels (n = 9 mice/group); (**L**) myocardial superoxide levels (n = 9 mice/group). ^*^P < 0.05 versus WT controls; ^†^P < 0.05 versus WT STZ groups; ^#^P < 0.05 versus Tg controls.

Oxidation of BH_4_ and dysregulation of NOS in diabetes are implicated in the pathogenesis of DCM^27^. We used high performance liquid chromatography (HPLC) to determine the concentrations of BH_4_ and Western blot to analyze the dimers, monomers, and phosphorylation of all 3 isoforms of NOS in the hearts of diabetic and non-diabetic Tg or WT mice. As shown in Fig. 6, diabetes significantly decreased cardiac BH_4_ concentrations, the ratio of nNOS dimers/monomers, phosphorylated nNOS (p-nNOS)/nNOS, eNOS dimers/monomers, and phosphorylated eNOS (p-eNOS)/eNOS and increased the expression of iNOS proteins in C57BL/6 WT mice compared with WT controls (P < 0.05 between WT STZ and WT controls, n = 4–5 hearts/group). GCH1 overexpression significantly increased cardiac BH_4_ concentrations and the ratios of nNOS dimers/monomers and p-nNOS/nNOS compared with WT controls. Interestingly, there were no significant differences in the ratios of nNOS dimers/monomers and p-nNOS/nNOS between Tg STZ and WT control groups (P > 0.05). Different from the effects of GCH1 overexpression on nNOS, GCH1 overexpression did not significantly alter the ratio of iNOS/GAPDH (glyceraldehyde 3-phosphate dehydrogenase) and p-eNOS/eNOS compared with WT controls. However, increased ratio of iNOS/GAPDH by diabetes was significantly reduced by GCH1 overexpression. Diabetes-induced decrease in the ratios of eNOS dimers/monomers and p-eNOS/eNOS were not significantly changed by GCH1 overexpression (P > 0.05 between Tg STZ and WT STZ groups, n = 4 hearts/group). Coupled NOS generates NO, whereas uncoupled NOS produces O_2_^•− ^22. Thus, we measured the levels of myocardial NO_x_ (tissue NO and its metabolite products, nitrate and nitrite) and O_2_^•−^. The production of NO_x_ and O_2_^•−^ was significantly elevated in WT STZ group compared with WT STZ groups. GCH1 overexpression normalized NO_x_ to the levels of WT control mice and significantly decreased the production of O_2_^•−^.

### GCH1 overexpression decreases phosphorylated p38 MAPK in diabetics

**Figure 7:**
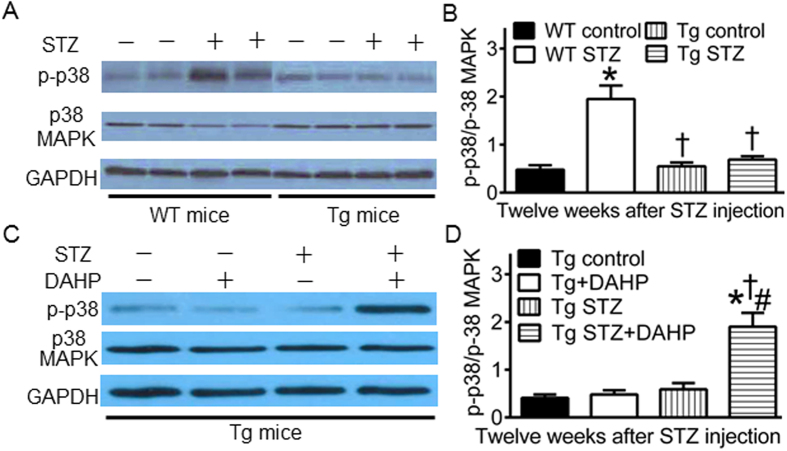
GTP cyclohydrolase 1 (GCH1) overexpression decreases phosphorylated p38 (p-p38) mitogen-activated protein kinase (MAPK) in diabetes. (**A**) representative Western blot bands showing the expression of cardiac p-p38 MAPK, p38 MAPK, and GAPDH proteins in mouse hearts; (**B**) reversal of ratio of p-p38/p-38 MAPK by GCH1 overexpression in diabetes. Wild-type (WT) and transgenic (Tg) mice were made diabetic with streptozotocin (STZ) for 12 weeks (WT STZ and Tg STZ groups), and control animals were given citrate buffer (WT control and Tg control groups). ^*^P < 0.05 versus WT control, ^†^P < 0.05 versus WT STZ, ^#^P < 0.05 versus Tg control (n = 4 hearts/group). (**C**) representatice Western blot bands showing the expression of p-p38 MAPK, total p-38 MAPK and GAPDH proteins in Tg mouse hearts with or without the treatment of 2,4-diamino-6-hydroxy-pyridine (DAHP); (**D**) increased ratio of p-p38 MAPK/p38 MAPK by DAHP in diabetic Tg mice. DAHP (a inhibitor for GCH1) was given for 4 weeks after 8 weeks of diabetes. ^*^P < 0.05 versus Tg control, ^†^P < 0.05 versus Tg + DAHP, ^#^P < 0.05 versus Tg STZ (n = 4 hearts/group).

Phosphorylated p38 (p-p38) MAPK is associated with cardiomyopathy^29^,^30^. We used Western blot to analyze the expression of p-p38 MAPK at tryptophan 180/tyrosine 182, p38 MAPK in Tg and WT mice with or without diabetes (Fig. 7). The ratio of p-p38 MAPK/p38 MAPK was significantly increased in WT STZ group compared with WT control (P < 0.05, n = 4 mice/group). The ratio of p-p38 MAPK/p38 MAPK was comparable between Tg control and WT control groups. Intriguingly, there were no significant differences in the ratio of p-p38 MAPK/p38 MAPK between Tg STZ and Tg control or WT control groups (P > 0.05).

To study if GCH1 contributes to a decrease in the ratio of p-p-38/p38 MAPK in diabetic Tg mice, we used DAHP to treat diabetic and nondiabetic Tg mice for 4 weeks. The ratio of p-p38 MAPK/p38 MAPK was not significantly altered by DAHP in nondiabetic GCH1-Tg mice (P > 0.05 between Tg + DAHP and Tg control groups, n = 4 mice/group). However, the ratio of p-p38 MAPK/p38 MAPK was significantly increased in Tg STZ + DAHP group compared with Tg control, Tg + DAHP, or Tg STZ group (P < 0.05).

### MG132 preserves GCH1 proteins and improves cardiac function in diabetic WT mice

**Figure 8:**
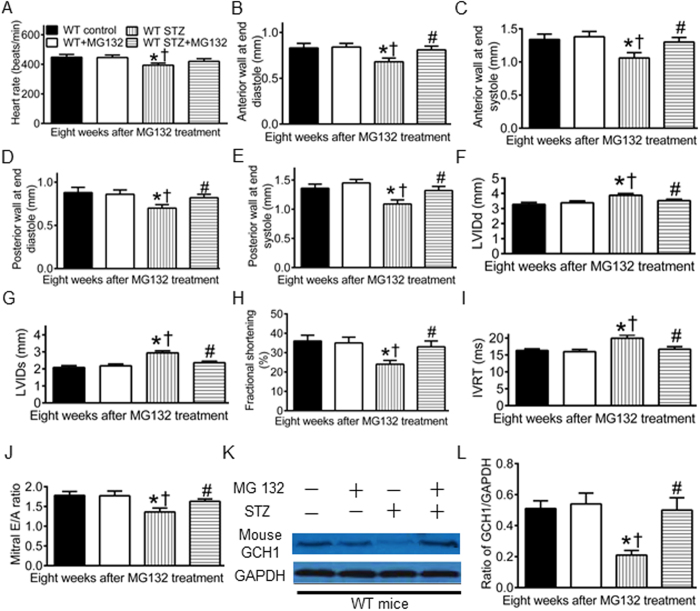
MG 132 ameliorates cardiac remodeling and dysfunction and preserves GCH1 proteins in diabetic wild-type (WT) mice. (**A**) heart rate; (**B**) anterior wall thickness at end diastole; (**C**) anterior wall thickness at end systole; (**D**) posterior wall thickness at end diastole; (**E**) posterior wall thickness at end systole; (**F**) left ventricular internal diameter at end diastole (LVIDd); (**G**) left ventricular internal diameter at end systole (LVIDs); (**H**) fractional shortening; (**I**) isovolumic relaxation time (IVRT); (**J**) mitral E/A ratio; (**K**) representative Western blot bands of mouse GCH1 and glyceraldehyde 3-phosphate dehydrogenase (GAPDH); (**L**) the ratio of GCH1/GAPDH. Diabetic and nondiabetic WT mice were given MG 132 for 8 weeks after 4 weeks of diabetes induced with streptozotocin (STZ) or vehicle as control. Echocardiography was used to assess the left ventricle of the mice. ^*^P < 0.05 versus WT controls; ^†^P < 0.05 versus WT + MG132; ^#^P < 0.05 versus WT STZ groups (n = 8–10 mice/group).

To study whether pharmacological approaches may preserve cardiac GCH1 proteins and diminish the severity of DCM, we used MG 132 to treat diabetic C57BL/6 mice for 8 weeks after 4 weeks of diabetes or nondiabetic C57BL/6 mice. The treatment of nondiabetic WT mice with MG 132 did not significantly alter the dimensions and function of the LV and ratio of GCH1/GAPDH (P > 0.05, n = 8–10 mice/group) (Fig. 8). Consistent with above results, diabetes caused decreases in wall thickness of the LV, fractional shortening, mitral E/A ratio, and ratio of GCH1/GAPDH and increases in LV internal diameters and IVRT 12 weeks after administration of STZ. Interestingly, these detrimental effects of diabetes were blocked by MG 132.

## Discussion

The results of the present study demonstrate that GCH1 is necessary for cardiac function in the mouse, however, cardiac GCH1 proteins are degraded in T1DM, leading to negative cardiac remodeling and dysfunction. Intriguingly, either cardiomyocyte-specific overexpression of GCH1 or inhibition of the 26S proteasome with MG 132 preserved cardiac GCH1 proteins and diminished diabetes-induced cardiac remodeling and dysfunction. GCH1-induced cardioprotection against DCM mainly involves the BH_4_/nNOS/SR Ca^2+^ handling proteins signaling pathway and depression of p38 MAPK in T1DM.

DAHP is a selective inhibitor of GCH1 and is often used to evaluate the function of GCH1^34^,^46^. In the present study, the treatment of WT mice with DAHP markedly elevated blood pressure and systolic and diastolic pressure of the LV. A previous study showed that the treatment of mice with DAHP resulted in eNOS uncoupling in vascular tissue due to deficiency of BH_4_^34^. Thus, DAHP-elicited increases in blood pressure and left ventricular pressure may be related to vascular endothelial dysfunction, that leads to hypertension^34^. In isolated Langendorff-perfused hearts, DAHP significantly decreased systolic pressure of the LV while the end-diastolic pressure was adjusted to the *in vivo* levels. Thus, GCH1 is necessary for cardiac function in the mouse.

The expression of cardiac GCH1 proteins was decreased by diabetes but the levels of GCH1 mRNA were not significantly altered. Therefore, diabetes-induced decreases in GCH1 proteins resulted from their degradation rather than a decrease in their biosynthesis. A previous study demonstrated that high glucose elevated the activity of 26S proteasome, leading to the degradation of vascular GCH1 proteins^20^. It is possible that decreased GCH1 proteins result from increased activity of 26S proteasome by diabetes. In the Tg mice, human GCH1 gene was used to increase GCH1 mRNA^32^. Interestingly, the total content of cardiac GCH1 proteins was more in the diabetic Tg mice than WT control mice. These results suggest that GCH1 transgene drives GCH1 protein expression in diabetic myocardium.

The present study kinetically monitored changes in the dimensions and function of the LV in WT and Tg mice following induction of diabetes. Diabetes resulted in significant decreases in the wall thickness and function of the LV and increases in LV volumes 12 weeks after induction of diabetes. GCH1 overexpression did not alter echocardiographic parameters and overall cardiovascular function of the mice, consistent with our previous report^32^. Interestingly, GCH1 overexpression reduced diabetes-induced remodeling and dysfunction of the LV. Moreover, GCH1 overexpression attenuated myocardial interstitial fibrosis (Fig. S9) and apoptotic cardiomyocytes (Fig. S10) induced by diabetes. Collectively, cardiomyocyte GCH1 may serve as a therapeutic target for DCM.

Our results indicated that GCH1 overexpression restored intracellular [Ca^2+^]_i_ and SR Ca^2+^ content that were decreased by diabetes. We also found that GCH1 overexpression increased the expression of RyR2 and SERCA2a proteins in nondiabetics and prevented diabetes-induced decreases in these two SR Ca^2+^ handling proteins. Thus, improved intracellular Ca^2+^ signaling by GCH1 overexpression in diabetes may be mainly attributed to up-regualation of SR Ca^2+^ handling proteins. In addition, GCH1 overexpression increased p-PLB at serine 16 in diabetics and nondiabetics. PLB is an inhibitory protein for SERCA2a, and its phosphorylation at serine 16 increases the affinity of SERCA2a for Ca^2+ ^47. Changes in p-PLB at serine 16 critically regulate the function of SERCA2a^48^. It is possible that increased p-PLB by GCH1 overexpression also contributes to improvements in intracellular Ca^2+^ signaling.

We demonstrated that diabetes decreased dimeric and phosphorylated nNOS and eNOS without altering the expression of total nNOS and total eNOS but increased the expression of total iNOS in WT mice. Interestingly, GCH1 overexpression eliminated diabetes-induced decreases in dimeric and phosphorylated nNOS and increases in iNOS but did not alter diabetes-induced reduction in dimeric and phosphorylated eNOS. Previously, we and other investigators have shown that cardiomyocyte-specific overexpression of GCH1 results in elevation of BH_4_ in cardiomyocytes rather than endothelial cells^19^,^33^. The selective effect of GCH1 overexpression on nNOS and iNOS may be related to the fact that nNOS and iNOS are mainly expressed in cardiomyocytes, whereas eNOS is predominantly expressed in vascular endothelial cells^49^,^50^.

Within cardiomyocytes, nNOS is localized in the SR and is physically linked with RyR2, PLB, and SERCA2a^51^. This enables nNOS to exert a direct effect on these proteins by NO from coupled nNOS or O_2_^•−^ from uncoupled nNOS. Caffeine-induced Ca^2+^ release is mediated by RyR2 in cardiomyocytes^52^. In the present study, GCH1 overexpression restored decreased RyR2-mediated Ca^2+^ release by diabetes, and the beneficial effects of GCH-1 overexpression on RyR2-mediated Ca^2+^ release were abrogated by S-methyl-L-thiocitrulline (SMTC, a specific inhibitor of nNOS) (Fig. S8). Therefore, GCH1 overexpression may exert favorable effect on RyR2-mediated Ca^2+^ release via nNOS in diabetes.

NO and O_2_^•−^ are the important mediator of myocardial protection and damage, respectively^53^,^54^. Diabetes elevated myocardial NO and O_2_^•−^ levels. Since nNOs and eNOS levels were decreased in diabetes, increased NO_*x*_ derived from elevated expression of iNOS. Previous studies have shown that iNOS-derived NO is able to cause myocardial damage^55^,^56^. This may be due to reaction of NO to high levels of O_2_^•−^ to form peroxynitrite in diabetes^57^,^58^. GCH1 overexpression decreased the expression of iNOS and O_2_^•−^ levels in diabetes. Thus, GCH1 overexpression is able to suppress oxidative stress in diabetes.

In the present study, diabetes increased p-p38 MAPK, and the negative effect of diabetes on p38 MAPK was suppressed by GCH1 overexpression. Furthermore, the effect of GCH1 overexpression on p38 MAPK was inhibited by DAHP. These results suggest that GCH1 overexpression can exert an inhitory effects on activation of p38 MAPK in diabetes. A growing body of evidence indicates that p38 MAPK regulates the expression and/or function of RyR2 and SERCA2a through direct or indirect (PLB) mechanisms^29^,^59^,^60^,^61^,^62^. We speculate that increased expression of RyR2 and SERCA2a proteins in diabetic Tg mice may be related to inhibiton of p-38 MAPK by GCH1.

We showed that MG 132 diminished the degradation of cardiac GCH1 proteins and reduced cardiac dysfunction in diabetes. MG 132 is a potent inhibitor for the 26S proteasome that is responsible for the degradation of GCH1 proteins^20^,^63^. Our present results suggest that inhibition of the 26S proteasome is a useful method to prevent the development of DCM. However, MG 132 may inhibit degradation of all classes of 26S proteasomal substrate rather than specific GCH1 proteins^64^. Recent progress on the study of proteasome reveals that the 26S proteasome can selectively recognize specific proteins for degradation through its 19S regulatory particle^65^. This property of the 26S proteasome suggests that there is a possibility to develop 26S proteasome inhibitors with specificity towards targeting proteins^66^. It is reasonably believed that developing novel 26S proteasome inhibitors specific for GCH1 proteins may be useful in the clinical treatment of DCM.

In summary, the present study indicates that degradation of cardiac GCH1 proteins contributes to the pathogenesis of DCM. Either cardiomyocyte-specific overexpression of GCH1 or inhibiton of the 26S proteasome with MG 132 protects the heart against DCM by elevating cardiac GCH1 proteins. The beneficial effects of GCH1 on diabetic hearts are associated with an improvement in intracellular Ca^2+^ signaling as a result of increases in BH_4_ bioavailability, dimeric and phosphorylated nNOS, and SR Ca^2+^ handling proteins and decreases in p-p38 MAPK and O_2_^•−^ production (Fig. S11). Our present study suggests that cardiomyocyte GCH1 is a potent therapeutic target for DCM, and developing novel 26S proteasome inhibitors with specificity towards cardiac GCH1 may be useful for the clinic treatment of DCM.

## Methods

### Animals

The Tg mice with cardiomyocyte-specific overexpression of human GCH-1 gene on a C57BL/6 background were generated, as described previously^32^. The Tg mice were identified by the presence of human GCH-1 gene using polymerase chain reaction (PCR) on tail-derived genomic DNA^32^. C57BL/6 WT littermates were used as control for the Tg mice. All protocols (Figs S1 and S2) were approved by the Animal Care and Use Committee of the Medical College of Wisconsin (Milwaukee, WI) and conformed to the Guide for the Care and Use of Laboratory Animals (Institute for Laboratory Animal Research, National Academy of Sciences, 8th edition, 2011).

### DAHP treatment of mouse

WT and Tg mice were orally given 50 mg/kg/day DAHP twice daily for 4 weeks (WT + DAHP and Tg + DAHP groups) using a plastic feeding tube or 1:1 dimethyl sulfoxide/NaCl (vehicle) as control (n = 10 mice/group)^34^,^46^.

### Determination of GCH1 activity

GCH1 enzyme activity was assessed as the conversion of GTP into 7,8-dihydroneopterin triphosphate, as described^19^.

### Induction of diabetes

T1DM was induced in Tg and WT mice at 6–8 weeks of age by daily intraperitoneal injection of 50 mg/kg/day STZ freshly prepared in citrate buffer (pH 4.5) for 5 consecutive days (WT STZ and Tg STZ groups)^20^. Control animals were given equivalent amounts of citrate buffer (WT control and Tg control groups).

### Administration of MG 132

Diabetic and nondiabetic C57BL/6 mice were injected intraperitoneally 10 μg/kg/day MG-132 for 8 weeks after 4 weeks of diabetes or equal amounts of vehicle as control^63^.

### Measurement of blood pressure, LV hemodynamics, and blood glucose

A Millar Mikro-Tip Pressure Transducer Catheter (Millar Instruments, Inc.) was inserted into the right carotid artery to monitor blood pressure and subsequently placed in the middle of the LV chamber to measure left ventricular systolic and diastolic pressure^35^. Fasting blood glucose of diabetic and nondiabetic mice was measured with a blood gas analyzer (ABL-725 Radiometer)^45^.

### Langendorff-perfused mouse hearts

Mouse hearts were mounted on a Langendorff apparatus and perfused retrogradely through the aorta at a constant pressure of 80 mmHg with Krebs-Henseleit buffer, as described^38^,^44^. The LV pressure signal, heart rate, and coronary flow rate were monitored, and +dP/dt (maximum rate of increase of left ventricular developed pressure) and −dP/dt (maximum rate of decrease of left ventricular developed pressure) were determined.

### PCR analysis

C57BL/6 mouse hearts were excised 0, 2, 4, 8, 12 weeks after administration of STZ, and the LV was homogenized at 4 °C for PCR analysis of mouse GCH-1 mRNA^33^,^44^.

### Transthoracic echocardiography

Echocardiography was performed with a VisualSonics Vevo 770 High-resolution Imaging System equipped with a 30 MHz transducer (Scanhead RMV 707), as described^39^,^45^. Left ventricular dimensions and ejection fraction were measured by two-dimension guided M-mode method. Pulsed Doppler waveforms recorded in the apical-4-chamber view were used for the measurements of the peak velocities of mitral E (early mitral inflow) and A (late mitral inflow) waves.

### Measurement of intracellular Ca^2+^

Cardiomyocytes isolated from adult mice were loaded with the fluorescence indicator fura-2 AM (5 μM) (F-1221, Molecular Probes) for 10 min at 22 °C, as described^44^,^67^. Basal [Ca^2+^]_i_, changes in [Ca^2+^]_i_ (∆[Ca^2+^]_i_), time to half (T50) decay of the Ca^2+^ transient, and the amplitude of the Ca^2+^ transient were measured in electrically stimulated (0.5 Hz) myocytes. SR Ca^2+^ content was assessed by rapid application of 10 mM caffeine to the cells to induce SR Ca^2+^ release in the presence of 0 Na^+^ and 0 Ca^2+^ Tyrode buffer to inhibit Na^+^ -Ca^2+^ exchange, as described^67^.

### Assay of biopterins

BH_4_ and 7,8-dihydroneopterin were quantified in LV tissue homogenates by HPLC with electrochemical detection (ESA Biosciences CoulArray® system Model 542), as described^68^,^69^. Authentic BH_4_ and 7,8-dihydroneopterin solutions (10–100 nM) were used as standards and sample concentrations were normalized to protein content measured by the bicinchoninic acid protein assay.

### Measurement of NO_x_ and O_2_^•−^

Tissue NO and its metabolite products (nitrate and nitrite) in the supernatant, collectively known as NO_*x*_, were assayed using a NO chemiluminescence analyzer (Siever 280i NO Analyzer)^32^,^70^. Lucigenin, a compound that emits light upon interaction with O_2_^•−^, was used to quantify the O_2_^•−^ production from myocardium, as described^71^. The data were presented in relative light units (RLUs) per mg protein. Relative O_2_^•−^ levels were expressed as percentages compared to WT controls.

### Immunoblotting

The effects of diabetes and GCH-1 overexpression on the expression of GCH-1 proteins, NOS and phosphorylated NOS proteins, SR Ca^2+^ handling proteins, and p38 MAPK were examined using standard Western blot techniques^44^,^70^. The normal function of nNOS and eNOS to produce NO requires dimerization of the enzyme^24^,^72^. To investigate nNOS and eNOS homodimer formation in the myocardium, non-boiled cellular lysate was resolved by 6% SDS-PAGE at 4 °C overnight^45^,^69^. The density of the Immunoreactive bands was analyzed with Image J (image acquisition and analysis software, NIH). In the Fig. 3, total GCH1 was the sum of human GCH1 band density and mouse GCH1 band density.

### Statistical analysis

All data are expressed as mean ± S.E.M. Statistical analysis was performed with one-way ANOVA followed by Bonferroni *post-hoc* test for multiple comparisons of multiple group means or with Student’s *t* test for comparisons between two group means. Repeated-measures ANOVA was used to compare the differences in body weight, blood glucose, heart rate, LV wall thickness, LV volumes, ejection fraction, mitral E/A ratio, GCH1 mRNA, GCH1 proteins, and ±dP/dt values at different time points. A value of P<0.05 was considered as statistically significant.

## Additional Information

**How to cite this article**: Wu, H.-E. *et al*. Cardiomyocyte GTP Cyclohydrolase 1 Protects the Heart Against Diabetic Cardiomyopathy. *Sci. Rep.* **6**, 27925; doi: 10.1038/srep27925 (2016).

## Supplementary Material

Supplementary Information
