# Supplementary material for: Cardiomyocyte GTP Cyclohydrolase 1 Protects the Heart Against Diabetic Cardiomyopathy
Source: Sci Rep. 2016 Jun 13;6:27925. doi: 10.1038/srep27925 (PMC4904741; doi:10.1038/srep27925)
Supplement: Supplementary Information [file srep27925-s1.pdf]

Supplementary Materials for

Cardiomyocyte GTP Cyclohydrolase 1 Protects the Heart Against Diabetic Cardiomyopathy

Hsiang-En Wu, Shelley L. Baumgardt, Juan Fang, Mark Paterson,

Yanan Liu, Jianhai Du, Yang Shi, Shigang Qiao, Zeljko J. Bosnjak,

David C. Warltier, Judy R. Kersten, Zhi-Dong Ge*

*Corresponding author. E-mail: Wilson.ge99@gmail.com

The Supplementary Materials includes:

Supplementary materials and methods
Fig. S1. Overall experimental protocol
Fig. S2. Experimental protocols for Western blot analysis
Fig. S3. Effects of GCH1 inhibitor on the hearts of WT and Tg mice
Fig. S4. Effects of diabetes on lung weight and tibia length in WT and Tg mice
Fig. S5. Effects of GCH1 overexpression on diabetic hearts in an isolated heart model
Fig. S6. Effects of nNOS inhibitors on cardiac function in Langendorff-perfused hearts
Fig. S7. Effects of sepiapterin on diabetic hearts of WT mice
Fig. S8. Effects of nNOS inhibitor on SR Ca^{2+} release in Tg mice
Fig. S9. Effects of GCH1 overexpression on fibrosis of diabetic hearts
Fig. S10. Effects of GCH1 overexpression on cardiomyocyte apoptosis of diabetic hearts
Fig. S11. Proposed mechanisms underlying protection of diabetic hearts by GCH1

Supplementary Materials and Methods

Animals

The transgenic (Tg) mice with cardiomyocyte-specific overexpression of human GTP cyclohydrolase 1 (GCH1) gene on a C57BL/6 background were generated under the control of the α-myosin heavy chain promoter, as described previously[1]. The Tg mice were identified by the presence of human GCH1 gene using polymerase chain reaction (PCR) on tail-derived genomic DNA[1,2]. C57BL/6 wild-type (WT) littermates were used as controls for the Tg mice.

The animals were kept on a 12-h light-dark cycle in a temperature-controlled room and received standard rodent maintenance diet and water *ad libitum*. All protocols (Figs. S1 and S2) were approved by the Animal Care and Use Committee of the Medical College of Wisconsin (Milwaukee, WI) and conformed to the Guide for the Care and Use of Laboratory Animals (Institute for Laboratory Animal Research, National Academy of Sciences, 8th edition, 2011).

2,4-diamino-6-hydroxy-pyridine (DAHP) treatment of mouse

WT and Tg mice were orally given 50 mg/kg/day DAHP (a specific inhibitor for GCH1) (Sigma-Aldrich, St. Louis, MO) twice daily for 4 weeks using a plastic feeding tube (Instech Laboratories, Inc., Plymouth Meeting, PA) or the vehicle, 1:1 dimethyl sulfoxide (DMSO)/NaCl, as control (n = 10 mice/group)[3,4]. Left ventricular diastolic and systolic pressure was determined with a pressure-tipped catheter placed within the chamber of the LV, as described in the section "Measurement of LV hemodynamics and blood glucose" below. Cardiac function was determined in Langendorff-perfused hearts or with echocardiography (Fig. S3), as described in the section "Transthoracic echocardiography" below.

Determination of GCH1 activity

GCH1 enzyme activity was assessed as the conversion of GTP into 7,8-dihydroneopterin triphosphate, as described[5]. Left ventricle (LV) was homogenized in lysis buffer and centrifuged. The supernatant was incubated with 50 μl of 10 mM GTP for 60 min at 37°C in the dark. Neopterins were quantified by comparison with the external standards after normalizing for sample protein content.

Induction of diabetes

Type 1 diabetes (T1DM) of insulin deficiency was induced in male Tg and C57BL/6 mice at 6-8 weeks of age by daily intraperitoneal injection of 50 mg/kg/day streptozotocin (STZ) (Cayman Chemical, Ann Arbor, MI) freshly prepared in 0.1 M sodium citrate buffer (pH 4.5) for 5 consecutive days[6,7]. Control animals were given equivalent amounts of sodium citrate buffer (pH 4.5).

Administration of MG 132

Diabetic and nondiabetic C57BL/6 mice were injected intraperitoneally 10 µg/kg/day MG-132 (Karebay™ Biochem, Inc., Monmouth Junction, NJ) for 8 weeks after 4 weeks of diabetes or equal amounts of vehicle (phosphate buffered saline [PBS] containing 0.0025 µg DMSO/ml)[8].

Measurement of LV hemodynamics and blood glucose

C57BL/6 and GCH1-Tg mice were fasted for 6 h and anesthetized by intraperitoneal injection of 80 mg/kg pentobarbital sodium (n=10 mice/group). The mice were ventilated with room air supplemented with 100% oxygen at approximately 102 breaths/min, as described[9]. A Millar Mikro-Tip Pressure Transducer Catheter (Millar Instruments, Inc., Houston, TX) was placed in the middle of the LV chamber via the right carotid artery[10]. The catheter was connected to a Powerlab data acquisition system (ADInstruments). After a 30 min of stabilization, the LV chamber pressure was continuously recorded for 20 min. A thoracotomy was performed, and the LV was punctured with a 27 gauge needle. Blood glucose was measured with a blood gas analyzer (ABL-725 Radiometer, Radiometer America Inc., Westlake, OH)[11]. Body temperature was maintained between 36.8 and 37.3°C throughout the experiment by using a heating pad (Model TC-1000, CWE Inc.; Ardmore, PA). After mice were euthanized, left ventricle and lung were weighed and normalized to body weight (Fig. S4).

Langendorff-perfused mouse hearts

Langendorff perfusion of mouse hearts. Pentobarbital-anesthetized mouse hearts were quickly excised at 4°C, mounted on a Langendorff apparatus, and perfused retrogradely through the aorta at a constant pressure of 80 mmHg with Krebs-Henseleit buffer containing (in mM) NaCl 118, $NaHCO_3$ 25, KCl 4.7, $MgCl_2$ 1.2, $CaCl_2$ 2.5, KH_2PO_4 1.2, EDTA 0.5, and glucose 11, as described[9,12]. The buffer was continuously bubbled with a mixture of 95% oxygen/5% carbon dioxide via an in-line filter (5 µm pore size). A fluid-filled plastic balloon was inserted into the chamber of the LV via the mitral valve, and connected to a pressure transducer for continuous measurement of LV pressure. The hearts were immersed in perfusate maintained at 37.2 ± 0.3^{o}C, and the balloon was inflated to a diastolic pressure of ~5 to 10 mmHg. Coronary flow was monitored by an in-line flow probe connected to a flow meter (Transonics Systems Inc., Ithaca, NY). The LV pressure signal was monitored, and +dP/dt (maximum rate of increase of left ventricular developed pressure) and –dP/dt (maximum rate of decrease of left ventricular developed pressure) were determined.

Experimental protocols. Langendorff-perfused hearts were used in two different protocols. (1) The effects of DAHP on cardiac function: WT and Tg mice were treated with DAHP for 4 weeks, as described in the section "2,4-diamino-6-hydroxy-pyridine **(**DAHP) treatment of mouse" above. Heart rate, cardiac function, and coronary flow rate were measured (Fig. S5). (2) The effects of nNOS on cardiac function in Tg mice. Cardiac nNOS regulates myocardial contractility and relaxation in normal hearts[5,13]. To study the direct role of nNOS in cardiac function, we determined the effects of S-methyl-L-thiocitrulline (SMTC, a specific inhibitor of nNOS) (Cayman Chemical) perfusion on myocardial contractility and relaxation in Langendorff-perfused hearts (Fig. S6). Diabetic and nondiabetic Tg hearts were stabilized for 30 min and

perfused with 100 nM SMTC or vehicle as control for 30 min. This utilization of SMTC has been indicated to inhibit the activity of nNOS in mice[5].

PCR analysis

C57BL/6 mouse hearts were excised 0, 2, 4, 8, 12 weeks after injection of STZ and snap frozen in liquid nitrogen immediately after excision. The LV was homogenized at 4 ^{o}C for PCR analysis of GCH1 mRNA[1,2]. Briefly, total RNA was extracted from snap frozen tissue in 1 ml of TRIzol reagent (Invitrogen, Carlsbad, CA) per 100 mg of heart tissue according to the protocol of the manufacturer. Genomic DNA was digested by treatment with RNase-free DNase (Ambion, Austin, TX), and RNA concentrations were determined spectrophotometrically. Complementary DNA was synthesized from 1 µg of total RNA and random hexamer primers by using SuperScript III first-strand synthesis system for PCR (Invitrogen) according to the manufacturer's directions, as described[1]. Complementary DNA was used in PCR with the following primers for various *GCH1* mRNA transcripts: human (transgenic): forward 5'-CGCCTACTCGTCCATCCTGA-3', reverse 5'- CCTTCACAATCACCATCTCA-3' (product size 181 bp); mouse (endogenous): forward 5'- TGCTTACTCGTCCATTCTGC-3', reverse 5'-CCTTCACAATCACCATCTCG-3' (product size 181 bp). Glyceraldehyde 3-phosphate dehydrogenase (GAPDH) was used as an internal control for all experiments. PCR reactions were performed in a 25-µl volume containing 1 µl of complementary DNA, 25 pmol of sequence-specific primers, and 22.5 µl of Plantinum PCR SuperMix High Fidelity (Invitrogen). A 10-µl aliquot of the PCR product was resolved by 1% Tris acetate-EDTA-agarose gel electrophoresis, and densitometric analysis of specific bands was performed using Alpha Imager (Alpha Innotech, San Leandro, CA). Relative mRNA level was determined as 2[(Ct/GAPDH − Ct/gene of interest)]. The results are presented as -fold expression normalized to GAPDH.

Transthoracic echocardiography

Echocardiographic examination of mouse. Mice were sedated by the inhalation of 1.5 % isoflurane and oxygen (n=12 mice/group). Non-invasive echocardiography was performed with a VisualSonics Vevo 770 High-resolution Imaging System (Toronto, Canada) equipped with a 30 MHz transducer (Scanhead RMV 707), as described previously[11,12,14]. Left ventricular dimensions and ejection fraction were measured by two-dimension guided M-mode method. Pulsed Doppler waveforms recorded in the apical-4-chamber view were used for the measurements of the peak velocities of mitral E (early mitral inflow) and A (late mitral inflow) waves.

Experimental protocols. Echocardiography was used in the following four protocols. (1) The effects of diabetes and GCH1 overexpression on the LV. C57BL/6 WT and Tg mice were made diabetic with STZ (WT STZ and Tg STZ groups), and control animals were given citrate buffer (pH 4.5) (WT control and Tg control groups). Echocardiography was used to evaluate the LV of the mice 0, 2, 4, 8, and 12 weeks after administration of STZ or citrate buffer (Fig. 4). (2) The effect of MG132 on diabetes-induced cardiac remodeling and dysfunction in WT mice. C57BL/6 mice were made diabetic with STZ for 4 weeks or given citrate buffer as control. Diabetic and diabetic WT mice were injected intraperitoneally MG 132 for 8 weeks. Echocardiography was used to assess the LV of the mice (Fig. 8). (3) The effects of GCH1 inhibition on cardiac function. WT and Tg mice were treated with DAHP for 4 weeks or 1:1 DMSO/NaCl (vehicle) as control (n=10 mice/group). Echocardiography was used to evaluate mouse hearts (Fig. S3). (4) The effects of sepiapterin (SEP) on diabetes-induced cardiac remodeling and dysfunction. BH_4 has been proposed to be a potential therapeutic target for cardiovascular disease[15,16]. To test the potential of BH_4 as a therapeutic target for DCM, we used SEP (a precursor of BH_4) to treat diabetic and nondiabetic WT mice[17,18]. The animals were orally given 5 mg/kg/day SEP for 8

weeks after 4 weeks of diabetes or vehicles as control (n=12-14 mice/group). The dimensions and function of the LV were assessed with echocardiography (Fig. S7).

Measurement of intracellular Ca^{2+}

Isolation of cardiomyocytes. Cardiomyocytes were isolated from adult mice, as described[12]. Briefly, mouse hearts were cannulated via the aorta onto a blunted 20 gauge needle and perfused for 10 min at 37 ^{o}C with perfusion buffer (in mM: 113 NaCl, 4.7 KCl, 0.6 KH_2PO_4, 0.6 Na_2HPO_4, 1.2 $MgSO_4$-$7H_2O$, 0.032 phenol red, 12 $NaHCO_3$, 10.0 $KHCO_3$, 10.0 HEPES, pH 7.4, 30 taurine, 10 2,3-butanedione monoxime, and 5.5 glucose) containing 0.25 mg/ml Liberase blendzyme I, 0.14 mg/ml trypsin, and 12.5 µM $CaCl_2$. After perfusion, the LVs were dissected free from the atria and repeatedly passed through a plastic transfer pipette to disaggregate the cells into a single-cell suspension. Subsequently, myocytes were enriched by sedimentation in perfusion buffer containing 5% bovine calf serum while slowly exposing the cells to increasing concentrations of $CaCl_2$ to achieve a final concentration of 1.2 mM. The final cell pellet containing calcium-tolerant myocytes was resuspended in the culture media containing Hanks' salts, 2 mM L-glutamine, 5% bovine calf serum, 10 mM 2,3-butanedione monoxime, and 100 U/ml penicillin. After isolation, the myocytes were stored in Tyrode solution (in mM: 132 NaCl, 10 HEPES, 5 glucose, 5 KCl, 1 $CaCl_2$, 1.2 $MgCl_2$; adjusted to pH 7.4). Experiments were conducted at room temperature within 5 h after isolation using Tyrode solution.

Measurement of intracellular [Ca^{2+}]. Cardiomyocytes isolated from adult mice were loaded with the fluorescence indicator fura-2 AM (5 µM) (F-1221, Molecular Probes) for 10 min at 22°C, as described[19]. After loading, myocytes were centrifuged, washed with normal Tyrode containing (mM): 140.0 NaCl, 5.0 KCl, 1.0 $MgCl_2$, 10.0 glucose, 5.0 HEPES, and 1.8 $CaCl_2$ (pH 7.4) to remove extracellular fura-2 and left for 30 min to ensure complete hydrolysis of the intracellular ester. To measure intracellular $[Ca^{2+}]_i$, cells were exposed to light emitted by a 150

W lamp and passed through either a 340 or a 380 nm filter. The resulting fluorescence emitted at 510 nm was recorded by a dual-excitation fluorescence photomultiplier (IonOptix LLC, Westwood, MA), and the ratio of the emitted fluorescence at the two excitation wavelengths (340/380 ratio) was calculated to provide an index of intracellular $[Ca^{2+}]_i$. Basal $[Ca^{2+}]_i$, changes in $[Ca^{2+}]_i$ ($\Delta[Ca^{2+}]_i$), time to half (T50) decay of the Ca^{2+} transient, and the amplitude of the Ca^{2+} transient were measured in electrically stimulated (0.5 Hz) myocytes.

Measurement of sarcoplasmic reticulum (SR) Ca^{2+} content. Cardiomyocytes were loaded with fura-2 AM with Pluronic F-127 (0.04%) to aid dispersion followed by 30 min of de-esterization at 22°C before recordings. Resting Ca^{2+} measurements were taken from the quiescent cells for 3-4 min prior to the application of any agonists. SR Ca^{2+} content was assessed by rapid application of 10 mM caffeine to the cells to induce SR Ca^{2+} release in the presence of 0 Na^+ and 0 Ca^{2+} Tyrode buffer to inhibit Na^+-Ca^{2+} exchange, as described [19].

Neuronal nitric oxide synthase (nNOS) is localized on the SR and physically linked with SR Ca^{2+} handling proteins[13, 20]. To study the correlation between nNOS activity and RyR2-mediated Ca^{2+} release (Fig. S8), cardiomyocytes isolated from diabetic or nondiabetic Tg mice were given 100 nM SMTC for 30 min or vehicle as control. Baseline Ca^{2+} measurements were taken on quiescent cells for 3-4 min prior to the application caffeine. The data in nNOS-inhibited cardiomyocytes were compared with those in vehicle-treated cells.

Assay of biopterins

Mouse hearts were rapidly excised 12 weeks after induction of diabetes or injection of citrate buffer (n=6 hearts/group). BH_4, BH_2, and 7,8-dihydroneopterin were quantified in LV tissue homogenates by high performance liquid chromatography (HPLC) with electrochemical detection (ESA Biosciences CoulArray® system Model 542, Chelmsford, MA), as previously described[17,21]. Filtrates were analyzed on a HPLC system (ESA Biosciences CoulArray®

system, Model 582 and 542) using an analytical Polar-RP column eluted with argon saturated 50 mM phosphate buffer (pH 2.6). Authentic BH_4, BH_2, and 7,8-dihydroneopterin solutions (10-100 nM) were used as standards and sample concentrations were normalized to protein content measured by the bicinchoninic acid protein assay.

Measurement of NO_x and $O_2^{\bullet-}$

LVs were homogenized and centrifuged at 14,000g for 20 min at 4°C. Tissue NO and its metabolite products (nitrate and nitrite) in the supernatant, collectively known as NO_x, were assayed using a NO chemiluminescence analyzer (Siever 280i NO Analyzer)[1].

Lucigenin, a compound that emits light upon interaction with $O_2^{\bullet-}$, was used to quantify the $O_2^{\bullet-}$ production from myocardium, as described[22]. Briefly, the LVs were homogenized in a HEPES-modified Krebs' buffer (in mM: 118.0 NaCl, 10.0 HEPES, 25.0 $NaHCO_3$, 5.6 glucose, 4.7 KCl, 1.2 KH_2PO_4, 1.1 $MgSO_4$, 1.4 $CaCl_2$, pH 7.4) containing a protease inhibitor cocktail (Roche Applied Science) and centrifuged. The supernatant was added to the reaction tube containing 5 µM of lucigenin followed by incubation for 1 min at 37°C. The light reaction between $O_2^{\bullet-}$ and lucigenin was detected in a 96-well microplate luminometer (GloMax, Promega) during the incubation. The data were presented in relative light units (RLUs) per mg protein. Relative $O_2^{\bullet-}$ levels were expressed as percentages compared to WT controls.

Immunoblotting

LVs were harvested and homogenized in a buffer containing 20.0 mM 3-(N-morpholino)propanesulfonic acid (MOPS), 2.0 mM EGTA, 5.0 mM EDTA, protease inhibitor cocktail (1:100; Calbiochem, San Diego, CA), phosphatase inhibitors cocktail (1:100;

Calbiochem), 0.5% detergent (Nonidet™ P-40 detergent pH 7.4, Sigma-Aldrich). Immunoblots were performed using standard techniques, as described[11,12,18]. Briefly, tissue homogenates that contained 50 μg of protein were applied to 7.5% sodium dodecyl sulfate (SDS)-polyacrylamide gel and subjected to immunoblot analysis by incubation with primary antibodies against human GCH1 (LifeSpan BioSciences, Inc., Seattle, WA), mouse GCH1 (BD Biosciences, San Jose, CA), nNOS (Invitrogen), phosphorylated nNOS (p-nNOS) (serine 1412, Affinity Bioreagents, Golden, CO), iNOS (Abcam, Cambridge, MA), eNOS (Santa Cruz Biotechnologies, Santa Cruz, CA), phosphorylated e-NOS (p-eNOS) (serine 1177, Cell Signaling), ryanodine receptor 2 (RyR2, EMD Millipore, Billerica, MA), SERCA2a (Abcam), total phospholamban (T-PLB, Abcam), phosphorylated PLB at serine 16 (p-PLB, Abcam), p38 MAPK (Cell Signalling, Danvers, MA), phosphorylated p38 MAPK (tryptophan180/tyrosine 182) (Cell signalling), and GAPDH (Abcam) at 4°C (Fig. S2). The membrane was washed and then incubated with the appropriate anti-mouse or anti-rabbit secondary antibody. The normal function of nNOS and eNOS to produce NO requires dimerization of the enzyme[23,24]. To investigate nNOS and eNOS homodimer formation in the myocardium, non-boiled cellular lysate was resolved by 6% SDS-PAGE at 4°C overnight[11,17]. Membranes were incubated with a 1:2,000 dilution of mouse anti-nNOS or anti-eNOS monoclonal antibody (BD Transduction Laboratories, San Jose, CA). Immunoreactive bands were visualized by enhanced chemiluminescence followed by densitometric analysis using image acquisition and analysis software (Image J, National Institutes of Health, Baltimore, MD).

Histological analysis

Hearts were excised from isoflurane-euthanized mice, washed in PBS, fixed overnight in 4% paraformaldehyde, and embedded in paraffin as described[25]. After serial sectioning of hearts (apex to base) 9–15, 5-μm sections were stained with Masson's trichrome. Fibrosis was stained

in blue, whereas cardiac myocytes were stained in red, as shown in Fig. S9. For detection of apoptotic cardiomyocytes, the sections were incubated for 1 h at 56°C, deparaffinized in xylenes, and rehydrated by an ethanol series[26]. Terminal deoxynucleotidyl transferase-mediated dUTP nick-end labeling (TUNEL) staining were performed with the use of the apoptosis detection Kits, DeadEnd™ Colorimetric TUNEL system, according to the manufacturer's instructions (Promega, Madison, WI) (Fig. S10). The slides were counterstained with hemotoxylin (blue).

References

1. Ge, Z. D. *et al.* Cardiac-specific overexpression of GTP cyclohydrolase 1 restores ischaemic preconditioning during hyperglycaemia. *Cardiovasc Res* **91**, 340-349 (2011).
2. Ionova, I. A. *et al.* Cardiac myocyte-specific overexpression of human GTP cyclohydrolase I protects against acute cardiac allograft rejection. *Am J Physiol Heart Circ Physiol* **299**, H88-H96 (2010).
3. Ceylan-Isik, A. F. *et al.* Metallothionein abrogates GTP cyclohydrolase I inhibition-induced cardiac contractile and morphological defects: role of mitochondrial biogenesis. *Hypertension* **53**, 1023-1031 (2009).
4. Pickert, G. *et al.* Inhibition of GTP cyclohydrolase reduces cancer pain in mice and enhances analgesic effects of morphine. *J Mol Med* **90**, 1473-1486 (2012).
5. Carnicer R*, et al.* Cardiomyocyte GTP cyclohydrolase 1 and tetrahydrobiopterin increase NOS1 activity and accelerate myocardial relaxation. *Circulation research* **111**, 718-727 (2012).
6. Like, A. A. & Rossini, A. A. Streptozotocin-induced pancreatic insulitis: new model of diabetes mellitus. *Science* **193**, 415-417 (1976).

7. Xu, J. *et al*. Proteasome-dependent degradation of guanosine 5'-triphosphate cyclohydrolase I causes tetrahydrobiopterin deficiency in diabetes mellitus. *Circulation* **116**, 944-953 (2007).
8. Wang, Y. *et al.* Therapeutic effect of MG-132 on diabetic cardiomyopathy is associated with its suppression of proteasomal activities: roles of Nrf2 and NF-kB. *Am J Physiol Heart Circ Physiol* **304**, H567-H578 (2013).
9. Ge, Z. D. *et al.* Cl-IB-MECA [2-chloro-N^6-(3-iodobenzyl)adenosine-5'-N-methylcarboxamide] reduces ischemia/reperfusion injury in mice by activating the A_3 adenosine receptor. *J Pharmacol Exp Ther* **319**, 1200-1210 (2006).
10. Liu, Y. *et al.* Inhibition of PKCβ2 overexpression ameliorates myocardial ischaemia/reperfusion injury in diabetic rats via restoring caveolin-3/Akt signaling. *Clin Sci* **129**, 331-344 (2015).
11. Baumgardt, S. L. *et al.* Chronic co-administration of sepiapterin and L-citrulline ameliorates diabetic cardiomyopathy and myocardial ischemia/reperfusion injury in obese type 2 diabetic mice. *Circ Heart Fail* 9, e002424 (2016).
12. Qiao, S. *et al.* MicroRNA-21 Mediates Isoflurane-induced Cardioprotection against Ischemia-Reperfusion Injury via Akt/Nitric Oxide Synthase/Mitochondrial Permeability Transition Pore Pathway. *Anesthesiology* **123**, 786-798 (2015).
13. Sears, C. E. *et al.* Cardiac neuronal nitric oxide synthase isoform regulates myocardial contraction and calcium handling. *Circu Res* **92**, e52-59 (2003).
14. Ge, Z. D. *et al.* Isoflurane postconditioning protects against reperfusion injury by preventing mitochondrial permeability transition by an endothelial nitric oxide synthase-dependent mechanism. *Anesthesiology* **112**, 73-85 (2010).
15. Moens, A. L. & Kass, D. A. Tetrahydrobiopterin and cardiovascular disease. *Arterioscler Thromb Vasc Biol* **26**, 2439-2444 (2006).

16. Bendall, J. K., Douglas, G., McNeil,l E., Channon, K. M. & Crabtree. M. J. Tetrahydrobiopterin in cardiovascular health and disease. *Antioxid Redox Signal* **20**, 3040-3077 (2014).

17. Vladic, N. *et al.* Decreased tetrahydrobiopterin and disrupted association of Hsp90 with eNOS by hyperglycemia impair myocardial ischemic preconditioning. *Am J Physiol Heart Circ Physiol* **301**, H2130-H2139 (2011).

18. Leucker, T. M. *et al.* Impairment of endothelial-myocardial interaction increases the susceptibility of cardiomyocytes to ischemia/reperfusion injury. *PloS One* **8**, e70088 (2013).

19. Al Kury, L. T. *et al.* Effects of endogenous cannabinoid anandamide on excitation-contraction coupling in rat ventricular myocytes. *Cell Calcium* **55**, 104-118 (2014).

20. Barouch, L. A. *et al.* Nitric oxide regulates the heart by spatial confinement of nitric oxide synthase isoforms. *Nature* **416**, 337-339 (2002).

21. An, J., Du, J., Wei, N., Xu, H., Pritchard, K. A. Jr. & Shi, Y. Role of tetrahydrobiopterin in resistance to myocardial ischemia in Brown Norway and Dahl S rats. *Am J Physiol Heart Circ Physiol* **297**, H1783-H1791 (2009).

22. Han, Y. *et al.* l-Tetrahydropalmatine, an active component of Corydalis yanhusuo W.T. Wang, protects against myocardial ischaemia-reperfusion injury in rats. *PloS One* **7**, e38627 (2012).

23. Forstermann, U. & Munzel, T. Endothelial nitric oxide synthase in vascular disease: from marvel to menace. *Circulation* **113**, 1708-1714 (2006).

24. Shang, T., Kotamraju, S., Kalivendi, S, V., Hillard, C. J. & Kalyanaraman, B. 1-Methyl-4-phenylpyridinium-induced apoptosis in cerebellar granule neurons is mediated by transferrin receptor iron-dependent depletion of tetrahydrobiopterin and neuronal nitric-oxide synthase-derived superoxide. J *Biol Chem* **279**, 19099-19112 (2004).

25. Teekakirikul, P. *et al.* Cardiac fibrosis in mice with hypertrophic cardiomyopathy is mediated by non-myocyte proliferation and requires Tgf-beta. *J Clin Invest* **120**, 3520-3529 (2010).

26. Li, B., Xiao, J., Li, Y., Zhang, J. & Zeng, M. Gene transfer of human neuregulin-1 attenuates ventricular remodeling in diabetic cardiomyopathy rats. *Exp Ther Med* **6**, 1105-1112 (2013).

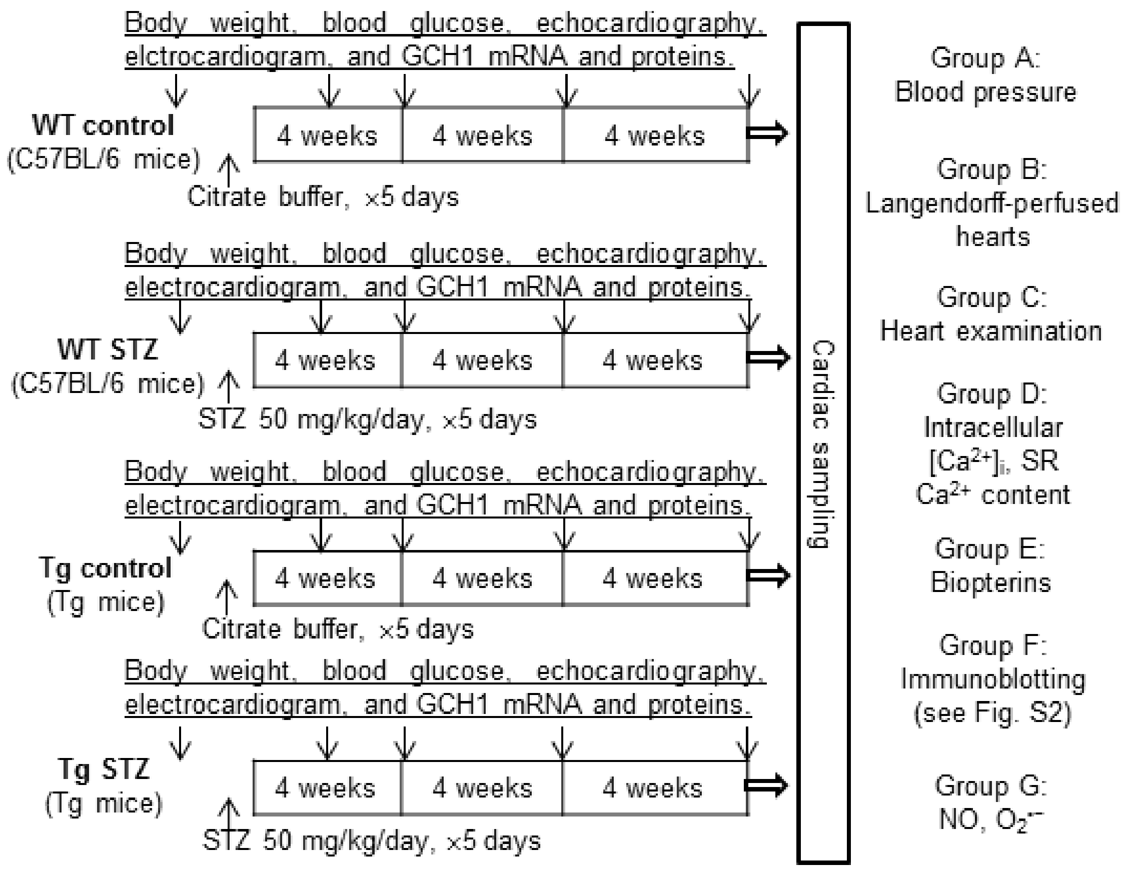

Figure S1 Schematic diagram depicting the overall experimental protocols.

Diabetes was induced in C57BL/6 wild-type (WT) mice and transgenic (Tg) mice with cardiomyocyte-specific overexpression of GTP cyclohydrolase 1 (GCH1) at 6-8 weeks of age by daily injection of 50 mg/kg/day streptozotocin (STZ) for 5 consecutive days. Control animals were given equivalent amounts of sodium citrate buffer (pH 4.5). Body weight, fasting blood glucose, the dimensions and function of the left ventricle, and GCH-1 mRNA and proteins were dynamically measured at baseline and 2, 4, 8, and 12 weeks after induction of diabetes. Blood pressure, heart weight, left ventricular weight, and lung weight were measured 12 weeks after induction of diabetes. Left ventricular tissues were sampling for measurements of intracellular Ca^{2+} signaling, biopetrins, nitric oxide (NO), superoxide ($O_2^{\bullet-}$), and Western blot analysis of GCH1, nitric oxide synthase, sarcoplasmic reticulum Ca^{2+} handling proteins, and p-38 mitogen-activated protein kinase.

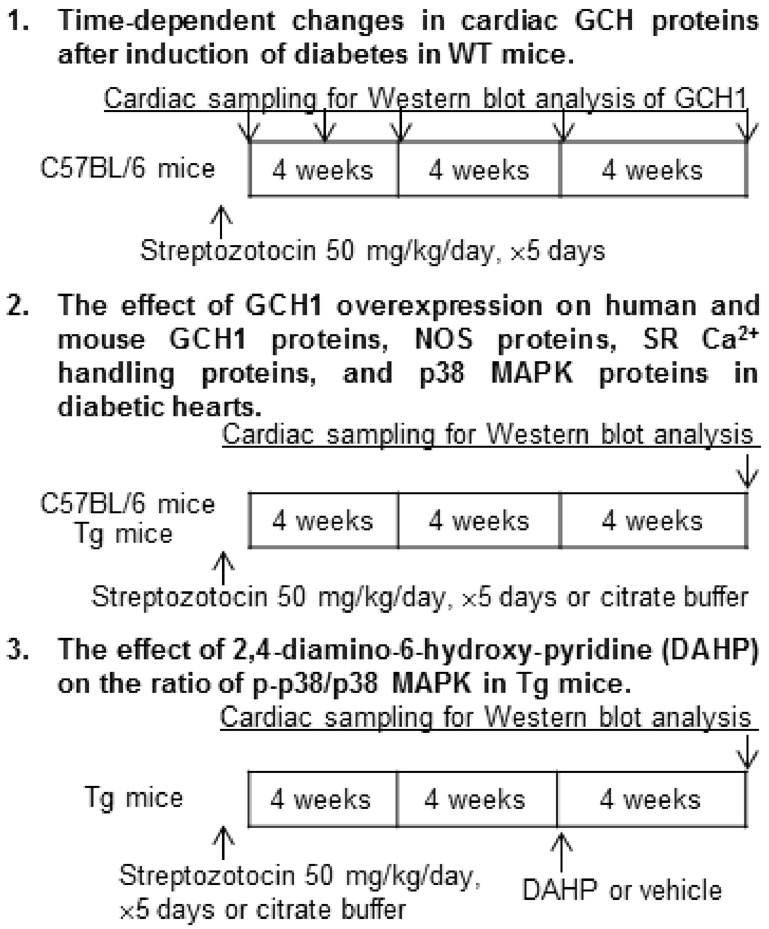

Figure S2 Schematic diagram depicting the experimental protocols for Western blot experiments.

Protocol 1: C57BL/6 wild-type (WT) mice at 6-8 weeks of age were given streptozotocin (STZ) to induce diabetes, and the hearts were harvested 0, 2, 4, 8 and 12 weeks after administration of STZ for Western blot analysis of GTP cyclohydrolase 1 (GCH1) proteins (n=4 mice/group). Protocol 2: C57BL/6 WT mice and Tg mice with cardiomyocyte-specific overexpression of GCH1 were administered STZ to induce diabetes or citrate buffer as control, and mouse hearts were collected 12 weeks after the injection for Western blot analysis of mouse and human GCH1 proteins, nitric oxide synthase (NOS) proteins, sarcoplasmic reticulum (SR) Ca^{2+} handling proteins, and p38 mitogen-activated protein kinase (MAPK) proteins (n=4 mice/group). Protocol 3: diabetic and nondiabetic Tg mice were orally given 50 mg/kg 2,4-diamino-6-hydroxy-

pyridine (DAHP) twice daily for 4 weeks started at 8 weeks after the injection of STZ or citrate buffer (n= 4 mice/group). The expression of p-38 MAPK and p-p38 MAPK was measured by Western blot analysis.

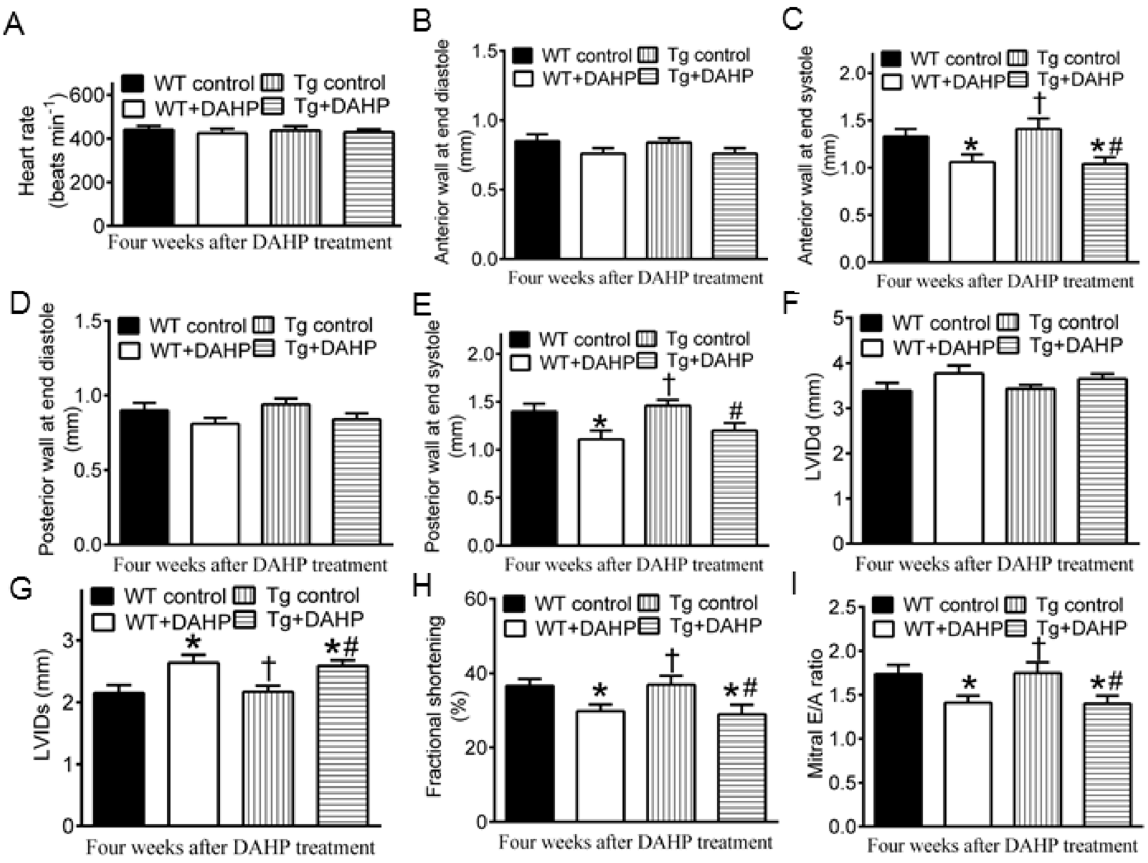

Figue S3 Inhibition of GTP cyclohydrolase 1 (GCH1) with 2,4-diamino-6-hydroxy-pyridine (DAHP) decreases systolic and diastolic function of both C57BL/6 wild-type (WT) and transgenic (Tg) mice.

A: heart rate; B: anterior wall thickness of the left ventricle at end diastole; C: anterior wall thickness of the left ventricle at end systole; D: posterior wall thickness of the left ventricle at end diastole; E: posterior wall thickness of the left ventricle at end systole; F: left ventricular internal diameter at end diastole (LVIDd); G: left ventricular internal diameter at end systole (LVIDs); H: fractional shortening; I: mitral E/A ratio. WT and Tg mice with cardiomyocyte-specific overexpression of GCH1 were given 50 mg/kg DAHP twice daily for 4 weeks or 1:1 dimethyl sulfoxide (DMSO)/NaCl as control. Echocardiography was used to evaluate the dimensions and function of the left ventricle. *P<0.05 versus WT controls; †P<0.05 versus WT+DAHP; $^{\#}$P<0.05 versus Tg controls (n=10 mice/group).

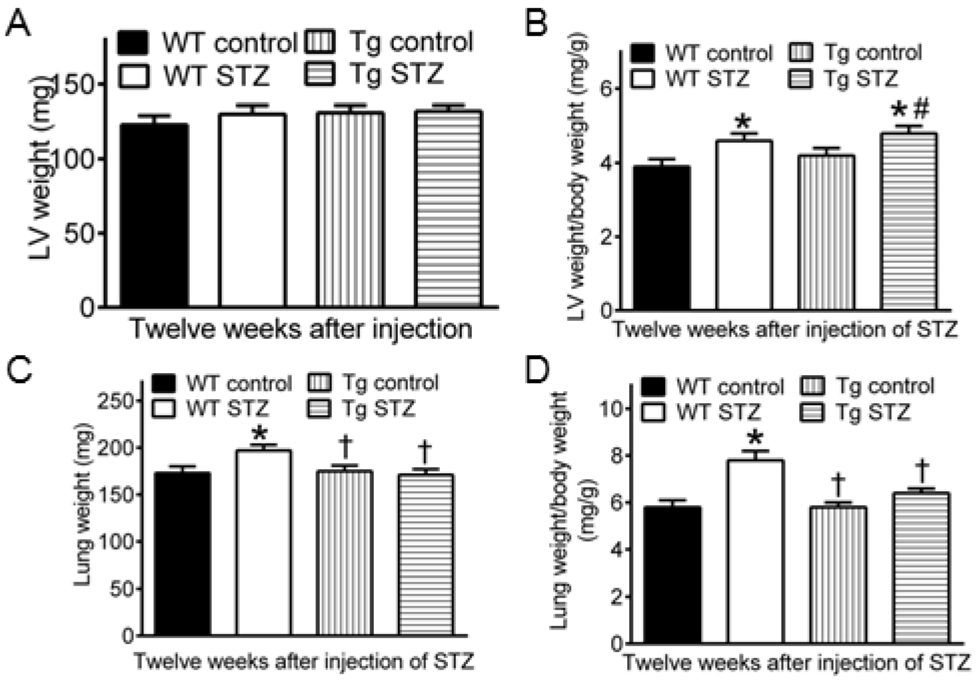

Figure S4 Cardiomyocyte-specific overexpression of GTP cyclohydrolase 1 (GCH1) decreases Lung weight and ratio of lung weight/body weight in diabetes but does not alter left ventricular (LV) weight and ratio of LV weight/body weight but

A: LV weight; B: ratio of LV weight/body weight: C: lung weight; D: ratio of lung weight/body weight. Wild-type (WT) and transgenic (Tg) mice were made diabetic with streptozotocin (STZ) or citrate buffer as control. LV and lung were weighted 12 weeks after induction of diabetes. . *P<0.05 versus WT controls; †P<0.05 versus WT STZ groups; #P<0.05 versus Tg controls (n=10 mice/group).

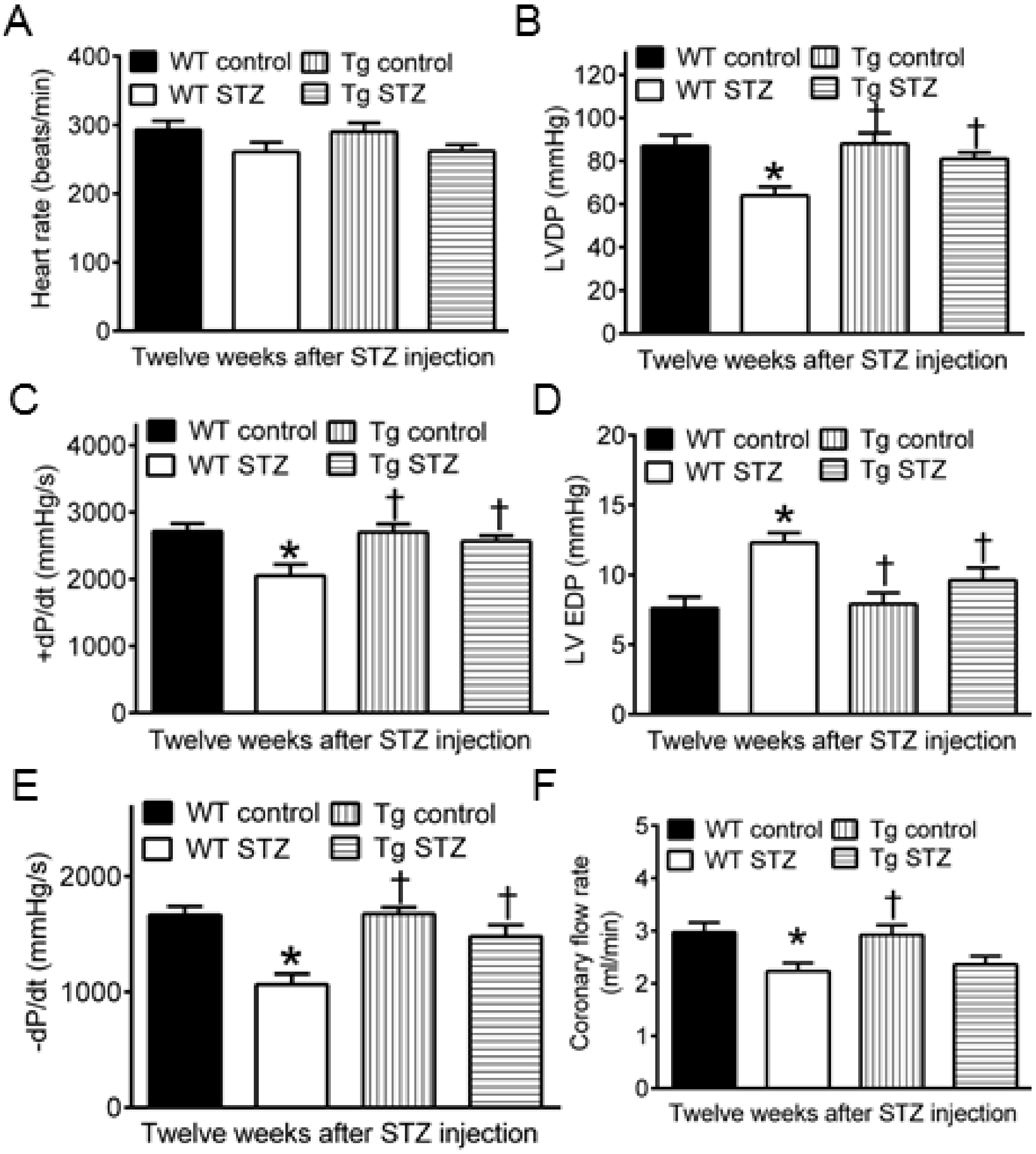

Figure S5 GTP cyclohydrolase 1 (GCH1) overexpression improves cardiac function impaired by diabetes in Langendorff-perfused hearts.

A: heart rate; B: left ventricular developed pressure (LVDP); C: +dP/dt (an index of systolic function); D: left ventricular end-diastolic function; E: -dP/dt (an index of diastolic function); F: coronary flow rate. Cardiac function was determined in Langendorff-perfused hearts isolated from wild-type (WT) and transgenic (Tg) mice with or without diabetes 12 weeks after

administration of streptozotocin (STZ) or vehicle. *P<0.05 versus WT controls; †P<0.05 versus WT STZ groups (n=10 hearts/group).

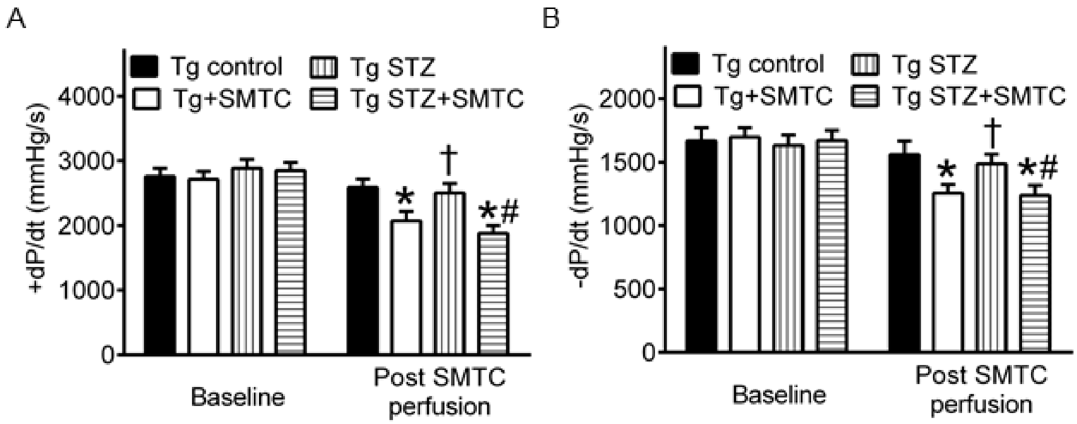

Figure S6 Inhibition of neuronal nitric oxide synthase (nNOS) decreases cardiac systolic and diastolic function in both nondiabetic and diabetic transgenic (Tg) mice with cardiomyocyte-specific overexpression of GTP cyclohydrolase 1 (GCH1)

A: +dP/dt (maximum rate of increase of left ventricular developed pressure, an index of left ventricular systolic function); B: -dP/dt (maximum rate of decrease of left ventricular developed pressure, an index of left ventricular diastolic function). Tg mice were made diabetic with streptozotocin (STZ) in Tg STZ and Tg STZ+SMTC (S-methyl-L-thiocitrulline, a specific inhibitor for nNOS) groups and given citrate buffer as control in Tg control and Tg+SMTC groups. The values of ±dP/dt were determined in Langendrff-perfused hearts after the hearts were equilibrated for 30 min and subsequently perfused with the buffer containing 100 nM SMTC or without SMTC for 30 min. *P<0.05 versus Tg control groups; †P<0.05 versus Tg+SMTC groups, $^{\#}$P<0.05 versus Tg STZ groups (n=8 hearts/group).

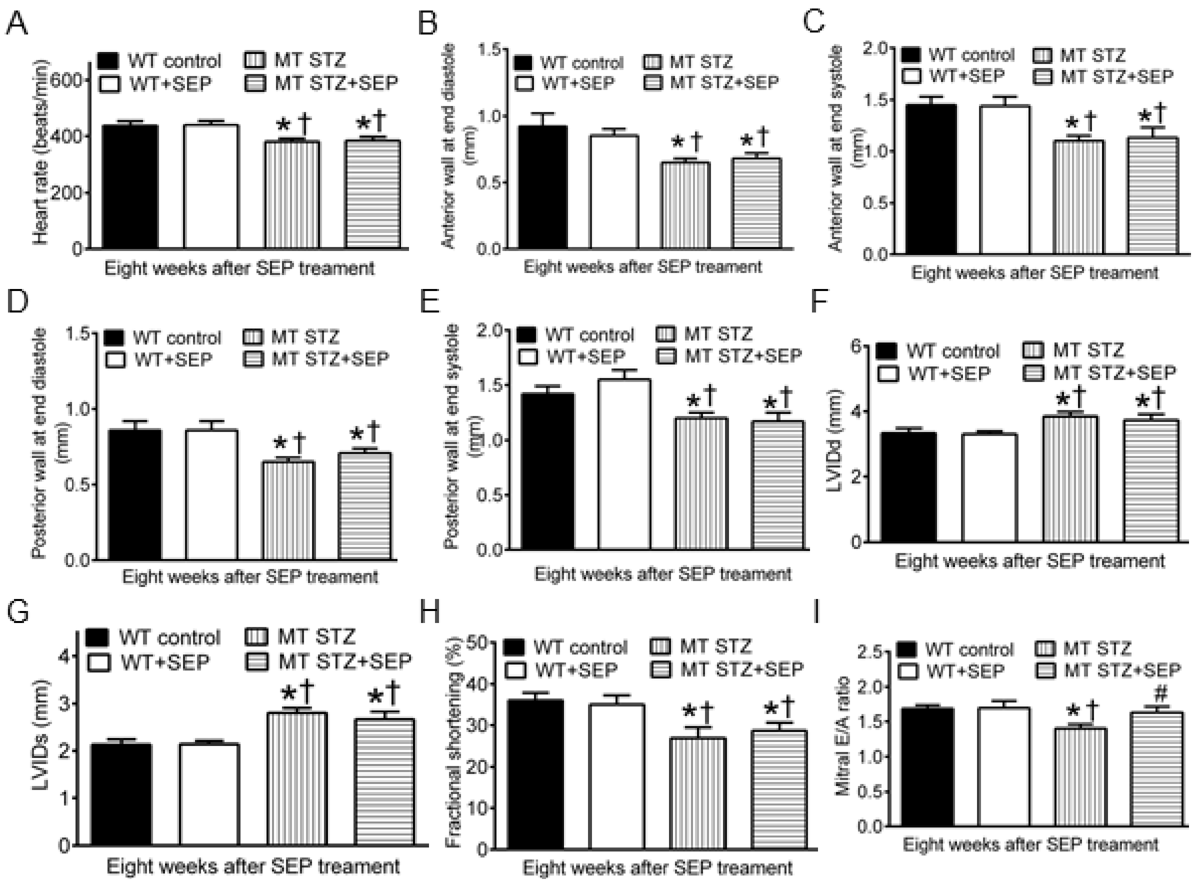

Figure S7 Sepiapterin (SEP) improves diastolic function of the left ventricle in diabetes but does not alter diabetes-induced cardiac remodeling and systolic dysfunction.

A: heart rate; B: anterior wall thickness of the left ventricle at end diastole; C: anterior wall thickness of the left ventricle at end systole; D: posterioa wall thickness of the left ventricle at end diastole; E: posterioa wall thickness of the left ventricle at end systole; F: left ventricular internal diameter at end diastole (LVIDd); G: left ventricular internal diameter at end systole (LVIDs); H: fractional shortening; I: mitral E/A ratio. SEP is a tetrahydrobiopterin precursor and is converted to tetrahydrobiopterin via the salvage pathway of tetrahydrobiopterin biosynthesis. Diabetic and nondiabetic WT mice were given orally 5 mg/kg/day SEP for 8 weeks after 4 weeks of diabetes induced with streptozotocin (STZ) or vehicle as control. Echocardiography

was used to assess the left ventricle of the mice. *P<0.05 versus WT controls; †P<0.05 versus WT+SEP; #P<0.05 versus WT STZ groups (n=12-14 mice/group).

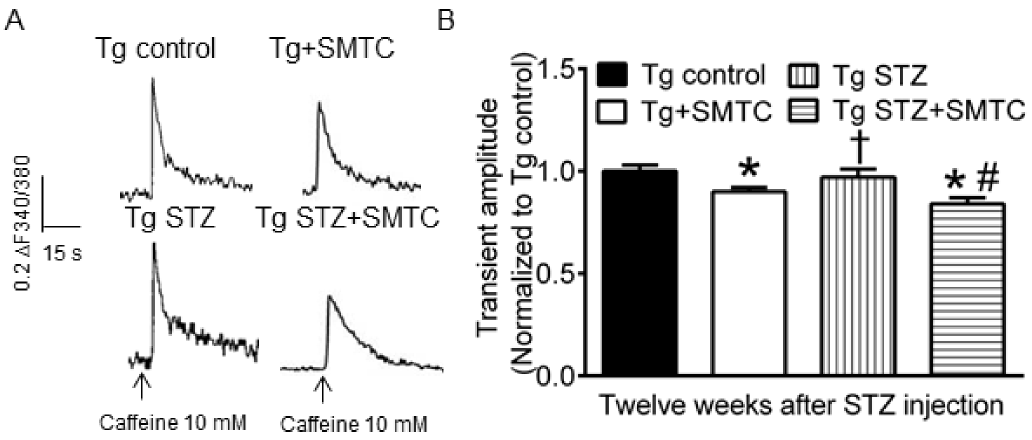

Figure S8 Neuronal nitric oxide synthase (nNOS) is involved in sarcoplasmic reticulum Ca^{2+} release in both nondiabetic and diabetic transgenic (Tg) mice with cardiomyocyte-specific overexpression of GTP cyclohydrolase 1 (GCH1)

A: original recordings of sarcoplasmic reticulum Ca^{2+} release induced by caffeine; B: inhibition of nNOS with S-methyl-L-thiocitrulline (SMTC, a specific inhibitor for nNOS) decreases caffeine-induced sarcoplasmic reticulum Ca^{2+} release in both nondiabetic and diabetic Tg mice. Cardiomyocytes were isolated from WT and Tg mice 12 weeks after induction of diabetes by STZ or citrate buffer as control. In Tg+SMTC and Tg STZ+SMTC groups, cardiomyocytes isolated from Tg control and Tg STZ mice, respectively, were incubated with SMTC for 30 min before application of caffeine. Arrows indicate that the application of caffeine to cardiomyocytes to induce Ca^{2+} release. *$P<0.05$ versus Tg control; $^{\dagger}P<0.05$ versus Tg SMTC group, $^{\#}P<0.05$ versus Tg STZ group (n=23-28 cells in 3 mice/group).

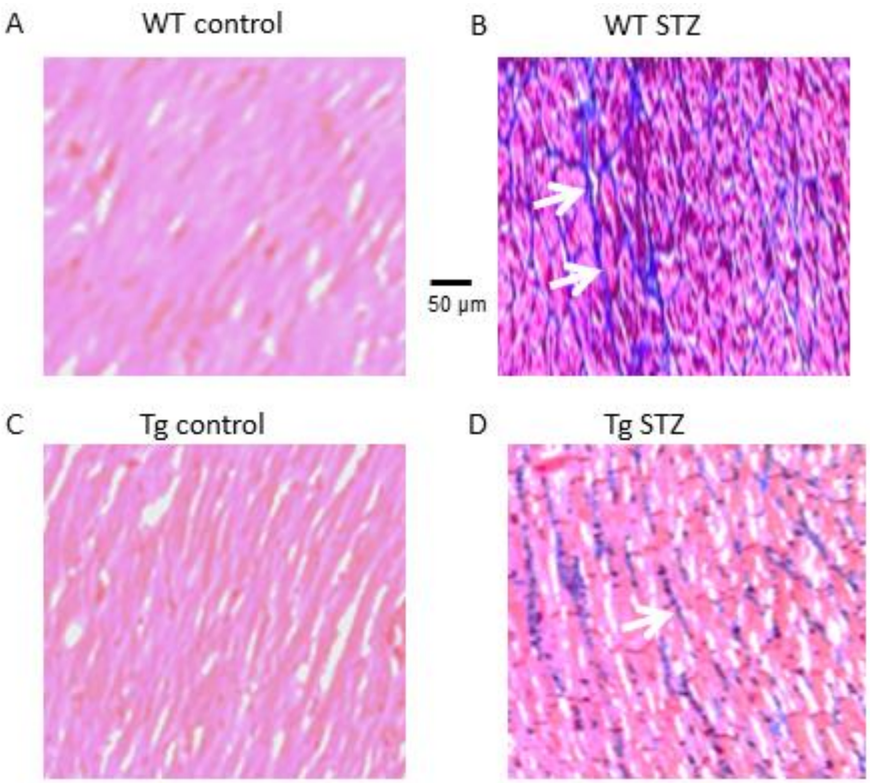

Figure S9 GTP cyclohydrolase 1 (GCH1) overexpression decreases interstitial fibrosis of diabetic hearts.

Representative photomicrographs of mouse hearts following a Masson's trichrome staining. Wild-type (WT) and transgenic (Tg) GCH1 mice were made diabetic with streptozotocin (STZ) (WT STZ and Tg STZ) for 12 weeks and citrate buffer as control (WT control and Tg control). Masson's trichrome was used to stain left ventricular sections from a WT control (A), a WT STZ (B), a Tg control (C), and a Tg STZ mouse (D). Interstitial fibrosis was stained in blue, as indicated by white arrows, and ventricular muscle was stained in red. The scale bar represents 50 µm.

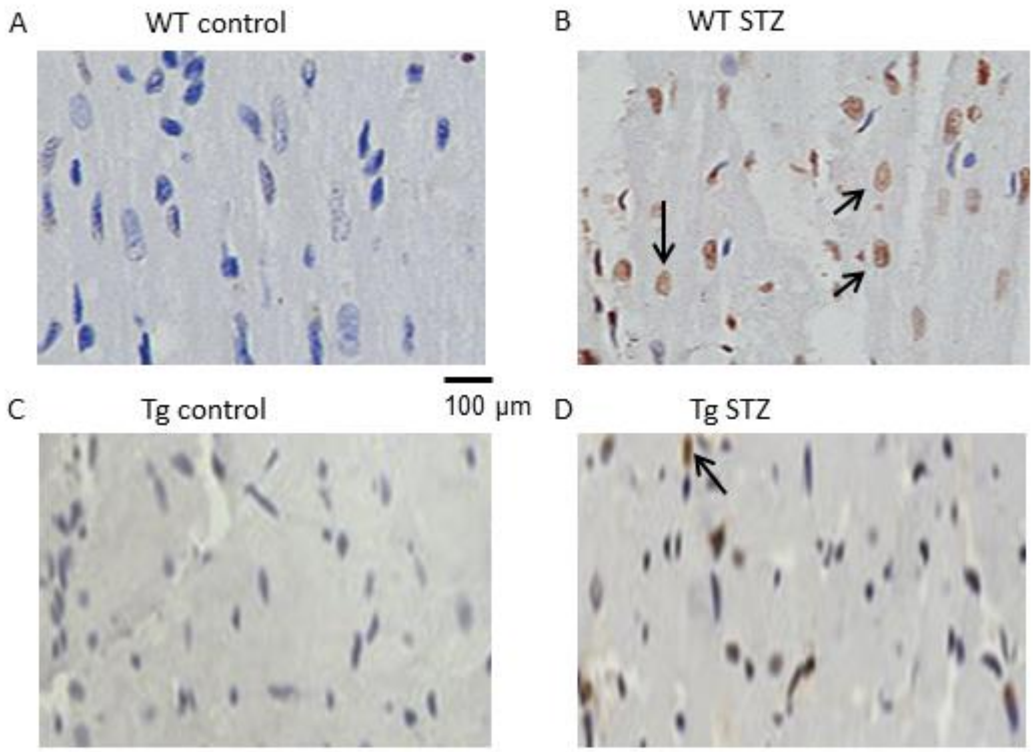

Figure S10 GTP cyclohydrolase 1 (GCH1) overexpression decreases cardiomyocyte apoptosis of diabetic hearts.

Representative photomicrographs of cardiomyocyte apoptosis following terminal deoxynucleotidyl transferase-mediated dUTP nick-end labeling (TUNEL) staining. Wild-type (WT) and transgenic (Tg) GCH1 mice were made diabetic with streptozotocin (STZ) for 12 weeks (WT STZ and Tg STZ) or citrate buffer as control (WT control and Tg control). Cardiomyocyte apoptosis was detected with a TUNEL assay. Black arrows show apoptotic cells (brown-stained cells). The scar bar represents 100 μm.

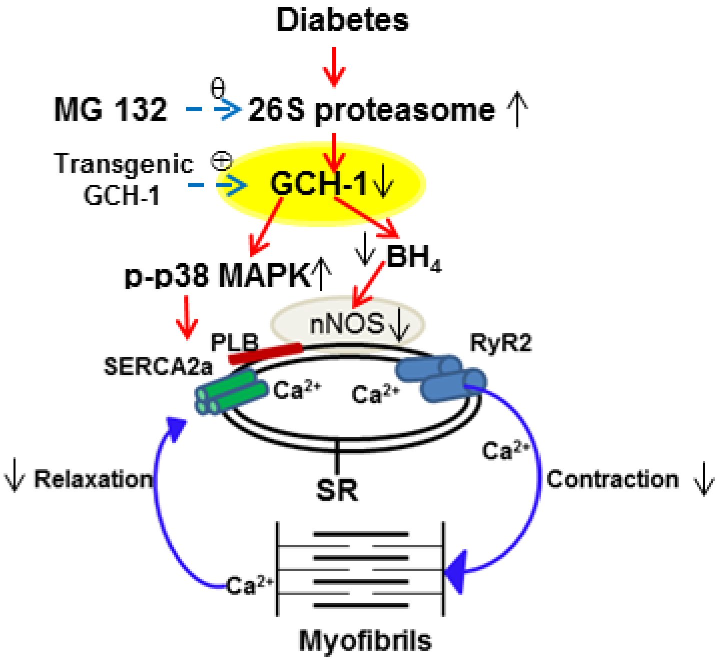

Figure S11 Proposed mechanisms responsible for GTP cyclohydrolase 1 (GCH1) modulation of myocardial contraction and relaxation in diabetes.

GCH1 is the first and rate-limiting enzyme in *de novo* biosynthesis of tetrahydrobiopterin (BH_4), a key co-factor for coupling of neuronal nitric oxide synthase (nNOS) to the substrate L-citrulline to produce the cadioprotective mediator, nitric oxide. Diabetes increases activity of 26S proteasome, which facilitates degradation of cardiac GCH1 proteins. Due to decreases in GCH1 and/or BH_4, nNOS localized in the sarcoplasmic reticulum (SR) becomes dysfunctional, and diabetes-induced increases in phosphorylated p38 (p-p38) mitogen-activated protein kinase (MAPK) are not inhibited, that causes decreases in ryanodine receptors (RyR2) and SR Ca^{2+} ATPase (SERCA2a) proteins. Dysfunctional nNOS and decreased RyR2 and SERCA2a together result in reduction in SR Ca^{2+} release via RyR2 and re-uptake of Ca^{2+} into the SR by SERCA2a. Thus, myocardial contraction and relaxation are depressed in diabetic cardiomyopathy. Cardiomyocyte-specific overexpression of GCH1 elevates cardiac GCH1 and abrogates the detrimental effects of diabetes on diabetic myocardium. MG 132 is a potent

inhibitor for the 26S proteasome and prevents the degradation of GCH1 proteins by the 26S proteasome in diabetes, thereby improving cardiac function.
